# Amyloid-Like Self-Assembling of Black Soldier Fly Proteins and Development of Bioplastics

**DOI:** 10.1021/acssuschemeng.5c07418

**Published:** 2025-10-18

**Authors:** Edoardo Testa, Elisa Fasoli, Paola Rizzo, Morena Casartelli, Gianluca Molla, Gianluca Tettamanti, Maurizio Galimberti

**Affiliations:** † 18981 Politecnico di Milano, Department of Chemistry, Materials and Chemical Engineering “G. Natta”, Via Mancinelli 7, 20131 Milano, Italy; ‡ Università degli Studi di Salerno, Department of Chemistry and Biology and INSTM Research Unit, Via Giovanni Paolo II 132, University of Salerno, 84084 Fisciano, Italy; § 9304 Università degli Studi di Milano, Department of Biosciences, Via Celoria 26, 20133 Milano, Italy; ∥ Università degli Studi dell’Insubria, Department of Biotechnology and Life Sciences, Via J. H. Dunant 3, 21100 Varese, Italy

**Keywords:** bioplastics, proteins, hermetia illucens, amyloid fibrils, packaging

## Abstract

The rising demand for sustainable materials has increased interest in biodegradable plastics. The black soldier fly (BSF) is a protein source characterized by the ability to thrive on organic waste, rapid development, and low environmental impact. This study shows that BSF proteins can undergo amyloid-like aggregation in alkaline environments, ultimately leading to amyloid fibrils suitable as reinforcing nanofillers for bioplastic films. The fibrillization process was monitored through Thioflavin-T (ThT) fluorescence assay and sodium dodecyl sulfate polyacrylamide gel electrophoresis (SDS-PAGE). The fibrils’ structure was studied by means of transmission electron microscopy (TEM) and one-dimensional/two-dimensional (1D/2D) X-ray diffraction (XRD) analyses performed on films obtained by casting. Bioplastic films were prepared by blending fibrillizated BSF proteins with poly­(vinyl alcohol) (PVOH) and glycerol. They exhibited thermal weldability and mechanical and gas barrier properties in line with those of the traditional oil- and biobased plastics used for packaging applications. Due to the current technological interest in BSF as a bioconverter of organic matter, the BSF protein-based materials presented in this work not only could help in mitigating the pressure arising from the accumulation of nonbiodegradable plastics but also provide tangible evidence about the valorization of municipal organic waste.

## Graphical abstract

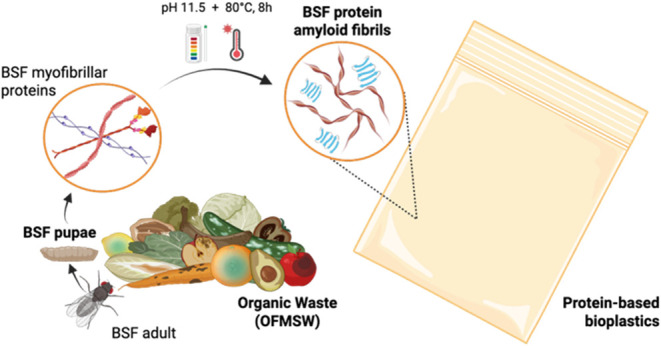

## Introduction

Since the 1950s, plastics have become one of the most important materials in our daily life: as of today, their cumulative amount accounts for 9.2 billion tons.^1^ The end of life of plastics represents a relevant problem for sustainable development: about 9–14% is recycled, the 12–14% is incinerated, and the vast majority (approximately 70–80%) ends up in landfills or is dispersed in the environment.^1–3^ The concerns associated with the environmental accumulation of nondegradable plastic waste and microplastics, i.e., any type of plastic fragments less than 5 mm in size,^4,5^ have thus accelerated the research and development of more sustainable alternatives. Bioplastics, especially those made from biodegradable polymers, are thus emerging as the preferred solution to mitigate the impact of traditional plastics through circular economy approaches.^6^

In this scenario, recent advancements in biopolymers research are underscoring the pivotal role of protein-based materials,^7–10^ especially when proteins are derived from waste organic feedstocks.^11^ Approaches aiming to prepare protein-based advanced materials span from genetically engineered proteins (i.e., recombinant proteins),^12,13^ to chemically cross-linked protein networks,^14–17^ and to self-assembled networks.^18–25^ In this scenario, the exploitation of protein’s self-assembly recently emerged as a viable and promising biomimetic method for its simplicity and environmental friendliness.

Amyloid fibrils are an example of a natural complex generated from the self-assembly of proteins.^26–28^ The study of these nanostructures has mainly focused on their association with neurodegenerative diseases such as Alzheimer’s and Parkinson’s diseases. Recent discoveries have shown additional potential for amyloid fibrils: they serve in natural structural roles, for example, in *Escherichia coli* biofilms, as catalysts in mammalian melanosomes and for hormone storage in humans.^22^ Their high physical and chemical stability has recently been exploited to develop high-performance bulk materials as alternatives to the traditional oil-based materials.^18,21,23,29–32^ For instance, Mezzenga et al. recently studied the transformation of various animal and plant proteins into amyloid structures, highlighting their potential in creating high-performance materials.^21,23,30,31,33^ Kamada et al. showcased a scalable method for creating, from fibril-like nanostructuring of soy and peas proteins, mechanically robust films with high optical transmittance and water stability.^18^ Complementary studies on amyloid fibrils from plant proteins and waste-derived feedstocks underscore both the versatility of fibril formation and its sensitivity to extraction chemistry and cocomponents and show that such fibrils can reinforce biodegradable matrices while enabling material functions (barrier/mechanics) comparable to petroleum-based plastics.^32,34–36^ These achievements pave the way for applications in a wide range of sectors from environmental remediation to disposable packaging and biomedicine.^22^

In this scenario, protein feedstocks can undermine sustainability when they compete with food supplies and demand land and water resources.^37,38^ This concern also applies to black soldier fly (BSF) proteins: indeed, recent studies have shown that BSF larvae can feed on various organic substrates to produce protein-rich meals.^39–41^ In this context, BSF proteins are currently being explored as novel and sustainable animal feed across the world.^42–48^ In this study, however, BSF proteins have been deliberately sourced from larvae reared on the organic fraction of municipal solid waste (OFMSW). Under EU guidance^49^ and EFSA recommendations, OFMSW-derived insect biomasses must be excluded from the food and feed chains due to microbiological risk. Thus, such proteins could represent a noncompeting, underutilized stream,^50^ and their valorization into functional bioplastics therefore advances circular economy goals without constraining BSF availability for food or feed.^50,51^ Notably, BSF was found as a valid alternative to composting or anaerobic digestion to treat the organic fraction of municipal solid waste (OFMSW), requiring a small amount of land and fresh water.^52,53^ In a recent paper, some of the authors reported that BSF can bioconvert the OFMSW into valuable biomacromolecules such as lipids, chitin, and proteins.^41^ From 1 kg of OFMSW, a total of about 50 g of BSF pupae, containing 6 g of proteins, 20 g of lipids, and 2 g of chitin, were obtained.^35,36,41^ Bioplastic films were recently obtained from a simple combination of BSF proteins and glycerol,^41^ evidencing significant improvements in their mechanical properties when compared to existing BSF protein films from the literature.^54–56^ BSF protein extracts were also employed as the main matrix components of novel electroconductive bionanocomposites for green flexible electronics.^57^ Despite these important advances in the valorization of BSF protein extracts, some improvements in mechanical, barrier, and thermal properties are still needed to enable wider application.

This work aimed to obtain amyloid fibrils from the protein extracts of BSF. The formation of amyloid fibrils from insect proteins is not documented in the literature and is a sought-after result.^29,58^ The protein extract was characterized by means of sodium dodecyl sulfate polyacrylamide gel electrophoresis (SDS-PAGE) and nanoliquid chromatography coupled to tandem mass spectrometry (nLC-MS/MS) analysis. The best conditions for the solubilization of BSF proteins were investigated, first by varying the pH of the solution. Fibrillization was then attempted by performing thermal annealing, monitoring the fibrillization process through the ThT fluorescence assay and SDS-PAGE. The protein’s structure was studied by means of transmission electron microscopy (TEM) and one-dimensional/two-dimensional (1D/2D) X-ray diffraction (XRD). Films were then prepared by casting a water solution of BSF proteins, poly­(vinyl alcohol) (PVOH), and glycerol, and the mechanical and gas barrier properties were investigated, analyzing the effect of the fibrillization procedure. A benchmark against the current materials for packaging applications is proposed. The work here reported is summarized in Figure 1.

**Figure 1:**
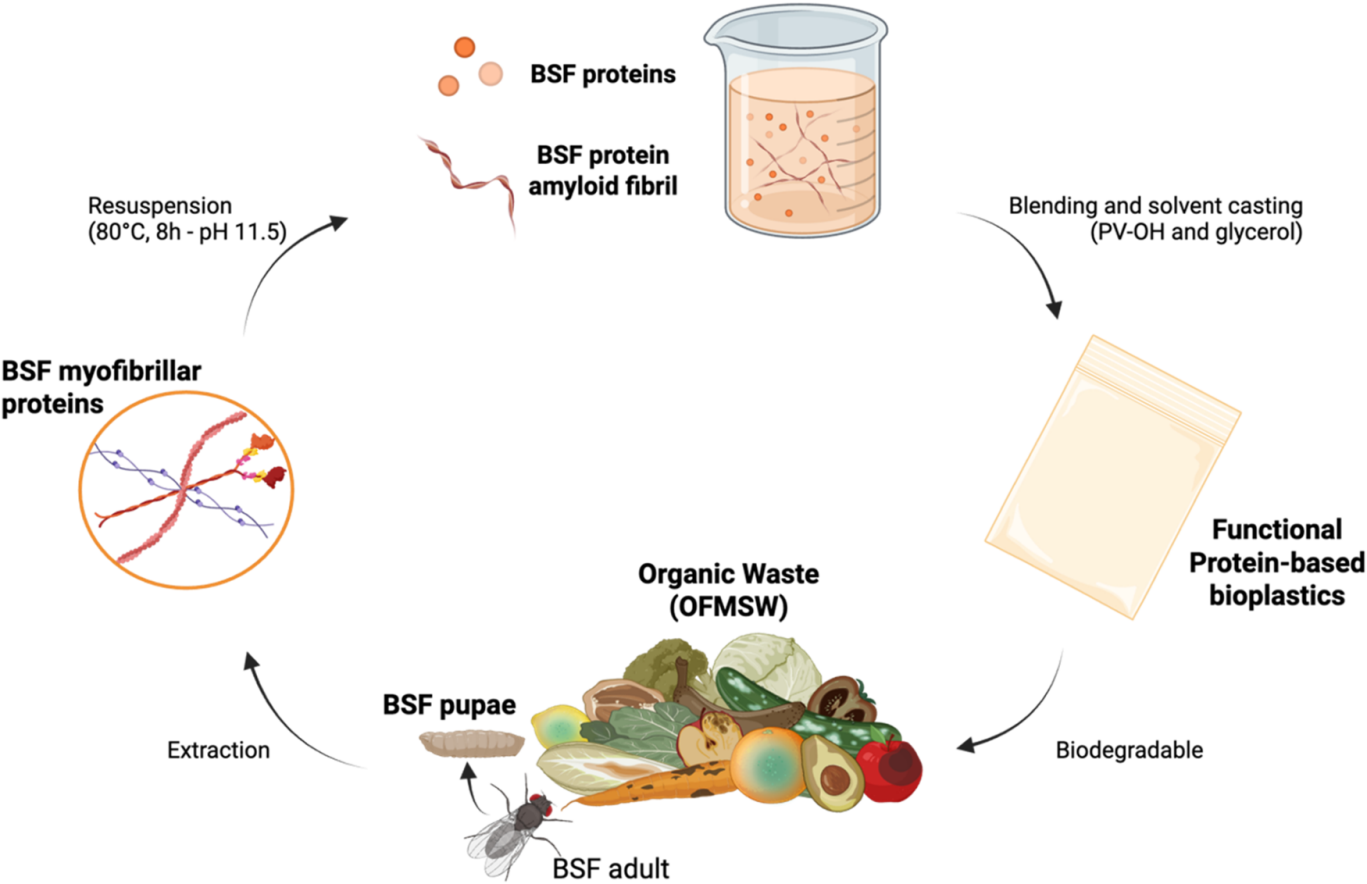
Production of biodegradable bioplastic films from proteins of BSF reared on the OFMSW. The process starts with the bioconversion of OFMSW mediated by the BSF, with proteins being extracted from the pupal stage of the insect as in a previous work.^41^ Given proteins, mainly myofibrillar, undergo ordered self-assembly in alkaline water solutions, evolving into amyloid fibril structures when exposed to prolonged heating at 80 °C. Through the addition of water-soluble and biodegradable ingredients (i.e., PV–OH and glycerol), high-performance and fully biodegradable bioplastic films are obtained [Copyright: Galimberti, M. – 2024, BioRender.com].

## Results and Discussion

### Origin and Main Features of BSF Proteins

The BSF proteins utilized in this study were obtained from the pupal stage of the insect *Hermetia illucens* reared on a surrogate OFMSW, whose composition was described in a recent work.^40^ Basically, the rearing substrate comprised a mix of bread, meat, cheese, fruits, and vegetables. The process leading to the extraction of the protein fraction from BSF meals, schematically shown in Figure S.1, was designed to balance process yield and protein purity, and it adhered to protocols reported by some of the authors in a recent publication.^41^ In brief, BSF pupae were freeze-dried, and meals were obtained by grinding. A defatting step was then performed in petroleum ether, followed by alkaline solubilization of the protein fraction and removal of chitin by centrifugation. Dried protein extracts were eventually obtained by isoelectric precipitation and pellet lyophilization.

The protein extract consisted of a mixture of proteins characterized by different molecular weights (MW), distributed within 10 and 250 kDa as from SDS-PAGE analysis (Figure S.2). Proteins were mainly of the myofibrillar type (data from nLC-MS/MS analyses, Table S.1), richer in tropomyosin, troponin, actin, and myosin, with few enzymes. These results reproduce those obtained in a previous work,^41^ where OFMSW with a slightly different nutritional composition was used.

### Acidic and Alkaline Solutions of BSF Protein Extracts

Protein fibrillization involves the self-assembly of misfolded or denatured proteins into highly ordered supramolecular structures.^22,59,60^ In this regard, protein solubility is critical in promoting the fibrillization process, as solubility determines the availability and stability of protein components, thus influencing the kinetics and thermodynamics of their assembling into fibrils.^27^ Factors affecting solubility, such as pH, ionic strength, protein concentration, and temperature, were thus considered.

The solubility in water of BSF protein extract was found to be dependent on pH as well as on the thermo-mechanical energy provided for preparing the solution. The effect of pH was evident when proteins were mixed with water under mild agitation (Figure S.3). At 0.1% w/v concentration, the lowest solubility of BSF protein extracts was observed around their isoelectric point (pH = 4.5, 12% soluble proteins from BCA assay). The solubility was low at pH 2 (30% of soluble proteins, BCA assay), while it increased for alkaline pH, reaching a maximum at pH 12 (85% soluble proteins, BCA assay). These results are in accordance with the used extraction method and are consistent with those reported in a previous work for protein extracts from BSF pupae reared on diets with slightly different nutritional compositions.^41^ Under the conditions of highest solubility (pH = 12), the dispersed particles exhibited the highest absolute value of the ζ potential (−31 mV). From TEM micrographs, aggregated particles with a size of approximately 50 nm were detected, constituted by 20–25 nm particles of spherical morphologies (Figure S.4).

Thermo-mechanical energy was provided by performing an ultrasonication treatment. Inspired by the work of Kamada et al.,^18^ BSF proteins were resuspended and studied in 0.1 M HCl (pH = 2), 5 M CH_3_COOH (pH = 3), and 0.1 M NaOH (pH = 11.5) water solutions. The turbidity of suspensions at 5% w/v protein concentration (as in previous studies for films preparation)^41^ was reduced, with a significant decrease in OD_600_ (Figures 2a and S.5). The solubility of BSF proteins was enhanced by the sonication step, at all of the tested pH values: pH = 2 (0.1 M HCl), pH = 3 (5 M CH_3_COOH), and pH = 11.5 (0.1 M NaOH). A solubility above 90% was achieved under each tested condition, with the highest values being obtained for alkaline protein suspensions (99%) (Figure 2b). It is worth mentioning that high solubility of raw protein extracts was reached even without sonication in 5 M CH_3_COOH. This result is in agreement with previous literature, where CH_3_COOH was chosen to enhance the solvation of hydrophobic amino acid residues under aqueous-compatible conditions.^18^

**Figure 2:**
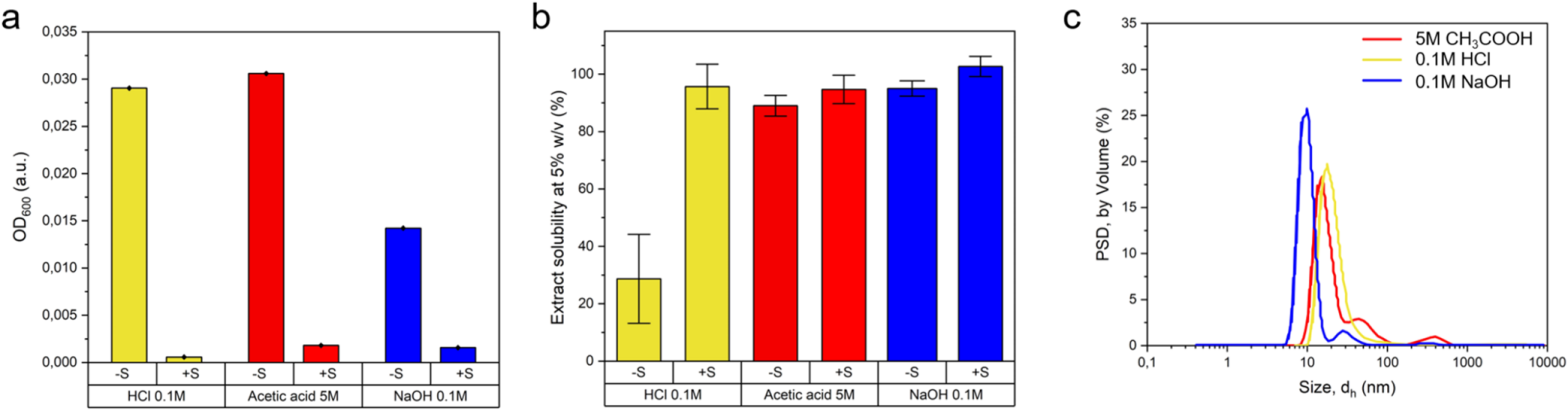
Dissolution and properties of BSF protein extracts in acidic and alkaline solutions. (a) BSF protein extract solubility at 5% w/v determined through centrifugal separation of nonsolubilized proteins for suspensions before (−S) and after (+S) the ultrasonication step (values are expressed as % of soluble matter on extract weight). Data are shown as mean ± s.d.; *n* = 3 (b) Turbidity measurements (OD_600_) for BSF protein extract suspensions (0.02% w/v) before (−S) and after (+S) the ultrasonication step. Data are shown as mean ± s.d.; *n* = 3. (c) Particle size distribution (PSD) by volume of BSF protein extract solutions after the ultrasonication step.

The ultrasonication treatment did not significantly alter the MW distribution of BSF proteins in either alkaline or acidic solutions but rather intensified band color in SDS-PAGE analyses (see Figure S.6). This suggests that sonication allowed more material to enter the gel, probably due to the overall increase in extract solubility. After sonication, BSF proteins displayed the lowest average particle size in an alkaline environment, i.e., 10 nm (compared to 18 nm for HCl, and 20 nm for CH_3_COOH) and the highest modulus of ζ-potential, i.e., −25 mV (compared to +15 mV for HCl and +11 mV for CH_3_COOH) (Figures 2c and S.7). The widest particle size distribution (PSD) was observed for 5 M CH_3_COOH protein solutions, with the biggest (few) particles observed in the range of dimensions between 300 and 1000 nm. PSD for 0.1 M HCl solutions was represented by Gaussian functions centered around 18 nm. 0.1 M NaOH protein solutions displayed the narrowest PSD. A small population of particles of 50 nm size was also detected, which probably caused few number fluctuations visible from the associated correlograms (Figure S.7).

### Film/Gel Formation from Alkaline and Acidic BSF Protein Extract Solutions

When mixed with glycerol (50% w/w, on proteins) and cast onto plasma desorption mass spectrometry (PDMS) substrates (see Experimental Section for more details), proteins from 0.1 M HCl and 5 M CH_3_COOH solutions rapidly formed gels (Figure 3a), which was not observed for proteins dissolved in 0.1 M NaOH. After solvent evaporation, only proteins from the 0.1 M NaOH solution led to the formation of homogeneous and free-standing films, while those from 0.1 M HCl and 5 M CH_3_COOH hardly formed continuous macroscopical structures (Figure 3b). Film fragments were formed from proteins suspended in 5 M CH_3_COOH, which showed an intense attenuated total reflectance-Fourier-transform infrared (ATR-FTIR) signal around 1620 cm^–1^ (Figure 3c, **red**), indicative of the aggregation between intermolecular β-sheets.^18,23,24,61,62^ A similar but less pronounced signal at 1620 cm^–1^ was observed in films from proteins suspended in 0.1 M HCl (Figure 3c, **yellow**), while films obtained from proteins suspended in 0.1 M NaOH led to the lowest ATR-FTIR signal around 1620 cm^–1^ (Figure 3c, **blue**). The weaker amide I band at ∼1620 cm^–1^ observed for NaOH-processed films is consistent with a slower evolution toward intermolecular β-sheets at high |ζ-Potential|, where electrostatic repulsion delays chain association during solvent removal. In contrast, acidic media (HCl/CH_3_COOH) promote fast β-sheet formation but lead to early gelation and poor film continuity.

**Figure 3:**
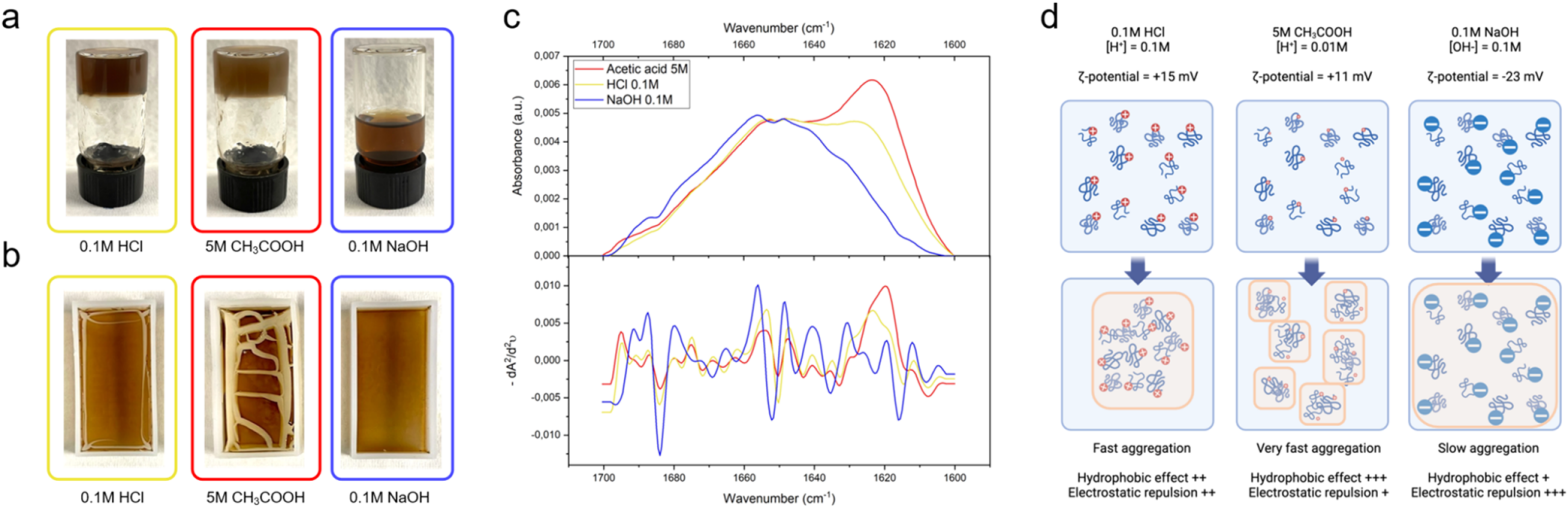
Film formation from acidic and alkaline BSF protein extract solutions. (a) Physical state of BSF protein extract solutions (5% w/v) 8 h after the ultrasonication step. (b) Visual appearance of BSF protein-based bioplastic films obtained after the casting process (48 h, 20 ± 2 °C). (c) ATR-FTIR absorbance spectra of the amide I region (1700–1600 cm^–1^) for BSF protein bioplastic films obtained from the three tested solvating conditions. On top: raw IR spectra (baseline corrected, normalized on signal at 1648 cm^–1^); on bottom: inverse of the second derivative of raw absorbance spectra (−dA^2^/d^2^υ) used to obtain relative secondary structure percentages by the second derivative band narrowing (SDBN) method. (d) The proposed mechanism underlying BSF protein aggregation and film formation for the tested solvating condition [Copyright: Galimberti, M. – 2024, BioRender.com].

For the sake of clarity, a mechanistic interpretation of BSF protein aggregation and film formation for each case study is illustrated in Figure 3d.

### From BSF Proteins to Amyloid Fibrils

The formation of amyloid fibrils from protein extracts, polypeptides, or peptides of animal or plant origin is documented in the scientific literature.^29,34,63^ While most protein fibrillization protocols reported for plant- and animal-derived proteins rely on acidic pH (usually pH = 2) to induce partial unfolding and promote β-sheet stacking,^23,29,34,36^ alkaline environments have also been reported to drive amyloid-like aggregation.^64–68^ In this regard, Ahmad et al. proposed that an alkaline pH may induce the formation of stable premelted globule states, which can be induced to aggregate into amyloid-like structures through hydrophobic interactions when the protein concentration is increased.^69^

In this work, the self-assembly of BSF protein extracts into amyloid fibrils was investigated with a special focus on alkaline solutions (0.1 M NaOH, pH 11.5). To induce fibrillization, BSF protein solutions were exposed to high temperature, i.e., *T* = 80 °C, from 0 to 24 h, and the fibrillization process was monitored through ThT fluorescence assays, SDS-PAGE, and TEM analyses on solutions prepared under film-forming conditions (i.e., *C*_protein_ = 5% w/v). Prolonged heating beyond 24 h resulted in increased hydrolysis and impaired film formation and was therefore not pursued.

After 8 h heating at *T* = 80 °C, the turbidity and color of BSF protein alkaline solutions significantly changed (Figure 4a), presumably due to thermally induced chemical degradation of pigments as well as deaggregation and/or hydrolysis of the protein components. This last hypothesis was validated through SDS-PAGE analyses on aliquots withdrawn from the solution at different heating times, from 0 to 24 h heating at *T* = 80 °C (Figure S.8). As detailed in Text S.1, the predominant band around 75 kDa disappeared after 2 h heating, while bands in the region between 50 and 37 kDa and below 20 kDa faded with the increase of the heating time. Partial hydrolysis of BSF proteins in alkaline environments has been documented in previous studies.^70^ At the same time, a pronounced increase in ThT fluorescence at 490 nm occurred, with the signal plateau achieved after about 6 h heating (Figure 4b). Notably, such a dependence of ThT fluorescence on time is similar to that reported for fibrillization experiments performed on plant proteins resuspended in acidic water solutions (pH 2) and heated at *T* = 90 °C for prolonged times.^23^ When left at rest (i.e., not exposed to *T* = 80 °C for prolonged times), BSF protein solutions gave a lower, but rather significant, ThT fluorescence signal, which remained unchanged over time up to 24 h at rest. Notably, when BSF proteins were exposed to hot acidic conditions (*T* = 80 °C, 0.1 M HCl), such a significant ThT fluorescence signal was not observed (Figure S.9).

**Figure 4:**
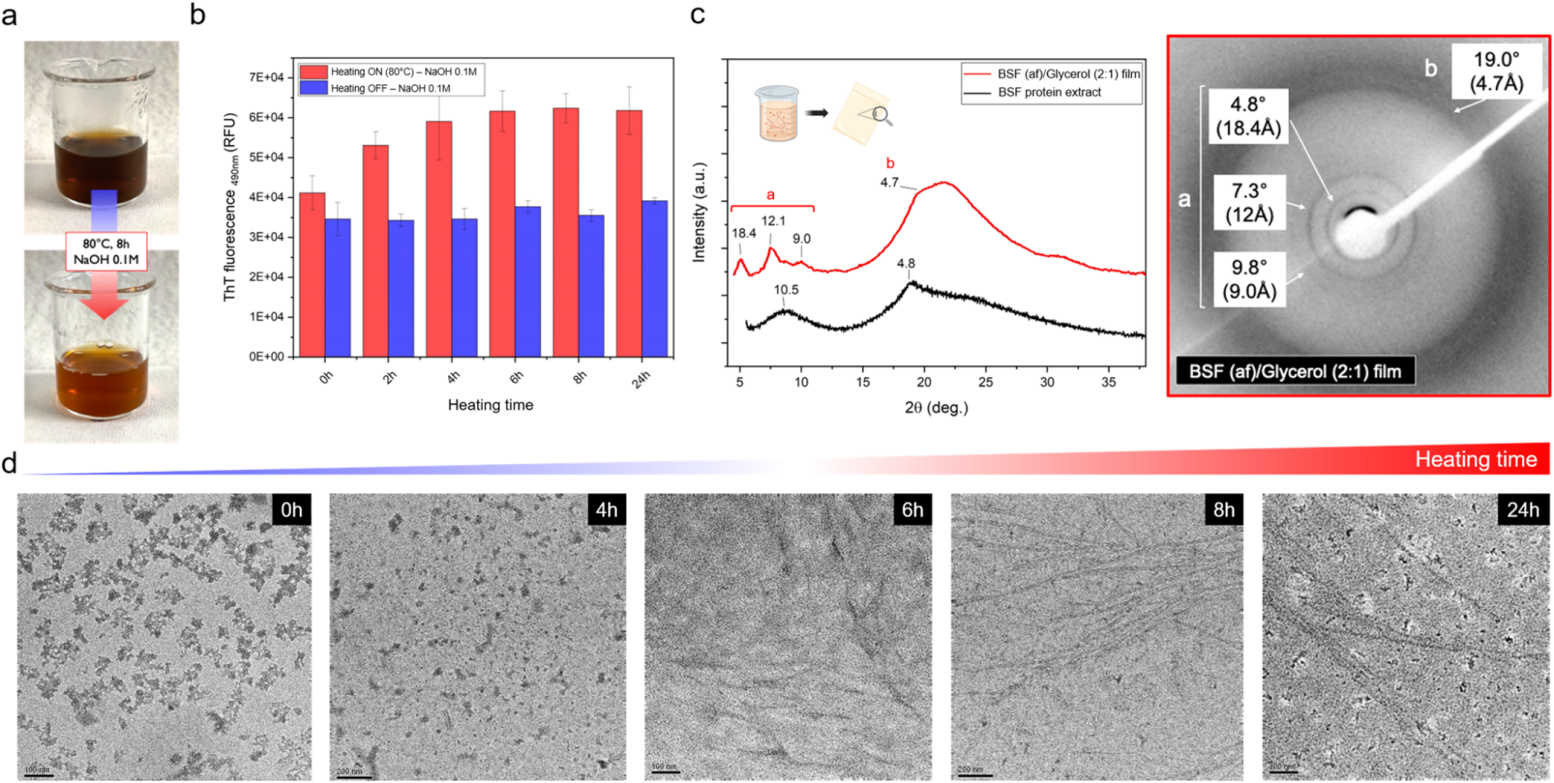
Transformation of BSF protein into amyloid fibrils. (a) Visual appearance of the alkaline BSF protein solution (5% w/v) before and after the fibrillization step. (b) ThT fluorescence emission at 490 nm for BSF protein solutions in 0.1 M NaOH exposed to 80 °C heating (red) or room temperature (blue). Solutions were prepared at a concentration of 5% w/v and diluted (1:20) at specified time steps in the ThT working reagent. Final protein concentration was 0.025% w/v. Data are shown as mean ± s.d.; *n* = 3. (c) Left: XRD powder pattern of starting BSF protein extract (black) and bidimensional XRD profile of BSF (af)/glycerol (2:1) film (red) [Copyright: Galimberti, M. – 2024, BioRender.com]. Right: bidimensional XRD image of a BSF (af)/glycerol (2:1) film. (d) TEM micrographs of 0.1 M NaOH suspensions of BSF protein exposed to different heating (80 °C) times (0, 4, 6, 8, 24 h). Starting samples (5% w/v) were diluted at desired time steps in Milli-Q water to 0.1% w/v and immediately transferred (6 μL) onto the carbon-coated copper grid for TEM analysis. Scale bars: 100 or 200 nm.

The formation of fibrillar structures in an alkaline environment was confirmed by TEM analyses (Figure 4d). In solutions heated at *T* = 80 °C, fibrillar aggregates began to form after 6 h, and mature long fibrils (up to μm in length) were observed after 8 h. After 24 h of heating, fibrillar structures were visible throughout the sample, along with many spherical particles not involved in the fibrillar aggregation. Such increased population of spherical aggregates, already observed in previous literature for some plant and algal proteins,^34,36^ may be consistent with concurrent alkaline hydrolysis (see Figure S.8), which generates shorter peptides prone to off-pathway association and oligomeric coacervation, thereby competing with fibril elongation. When not exposed to heating, the elements in solution were seen to consist of amorphous aggregates at each time point tested (Figure S.10).

For the sake of simplicity, herein, after the process of forming such fibrillar aggregates from BSF protein solutions in a hot alkaline environment (0.1 M NaOH, *T* = 80 °C, 8 h heating) will be named the “*fibrillization step*”, and the obtained fibrillizated proteins will be named *BSF­(af)*.

To investigate the solid-state organization of the fibrillar aggregates, thin films were prepared by casting a water solution of fibrillizated BSF proteins and glycerol (2:1), and bidimensional XRD analyses were performed (Figure 4c). For comparison, the powder XRD pattern of the pristine BSF protein extract was also reported in Figure 4c. XRD is a key method for analyzing the molecular arrangement of protein components in supramolecular aggregates, as amyloid fibrils.^29,71–75^ In particular, the hallmark of amyloid fibrils is their crystalline cross-β-sheet architecture, where β-strands run perpendicular to the fibril axis and the β-sheets stack along the fibril’s length. This gives rise to a periodicity of about 4.7 – 4.8 Å (2θ ≈ 19°) for adjacent β-strands and of 6–12 Å (2θ ≈ 7–15°) for the β-sheets stacking along the fibril axis.^29^ Both reflections are clearly visible in the XRD bidimensional profiles and patterns of the analyzed film (reflections *a* and *b* in Figure 4c). Particularly, beside the most intense peak at 2θ–19.0° corresponding to a spacing of 4.7 Å (attributed to the distance between hydrogen-bonded-β strands within β-sheet), narrow peaks at 2θ–4.8, 7.3, and 9.8° associated with spacings of 18.4, 12.1, and 9 Å were noticed. Such reflections, not observed in the pristine BSF protein extract, indicate a high level of crystalline order associated with large crystallites, typical only of a few types of amyloid fibrils originating from the regular stacking of short peptides.^74–77^

In light of the obtained results and considering the existing literature on the topic, such fibrillization of BSF proteins can be explained as follows. At a strong alkaline pH (i.e., pH = 11.5), the high net negative charge on polypeptide chains enhances electrostatic repulsion, preventing premature random aggregation and allowing the protein components to remain highly dispersed in solution, as evidenced in the previous paragraphs of this paper. Upon prolonged heating, partial alkaline hydrolysis follows, producing low-MW peptide fragments with increased chain mobility, which can more easily align into intermolecular β-sheets. Notably, this behavior has been observed in milk and legume protein systems, where fibril formation at elevated pH has been reported to depend on peptide bond cleavage and modified electrostatic environments—mediated by chaotropic or reducing agents—to enable β-sheet stacking and nanofibril assembly.^78^

### Films Based on Fibrillizated BSF Proteins, PVOH, and Glycerol

Bioplastic films from BSF protein were obtained through the process outlined in Figure 5 (see the Experimental Section for further details). Briefly, after protein dispersion, sonication, and fibrillization, glycerol and PVOH were added to the solution. Films were eventually obtained by casting the physical mixture into PDMS molds, followed by solvent evaporation.

**Figure 5:**
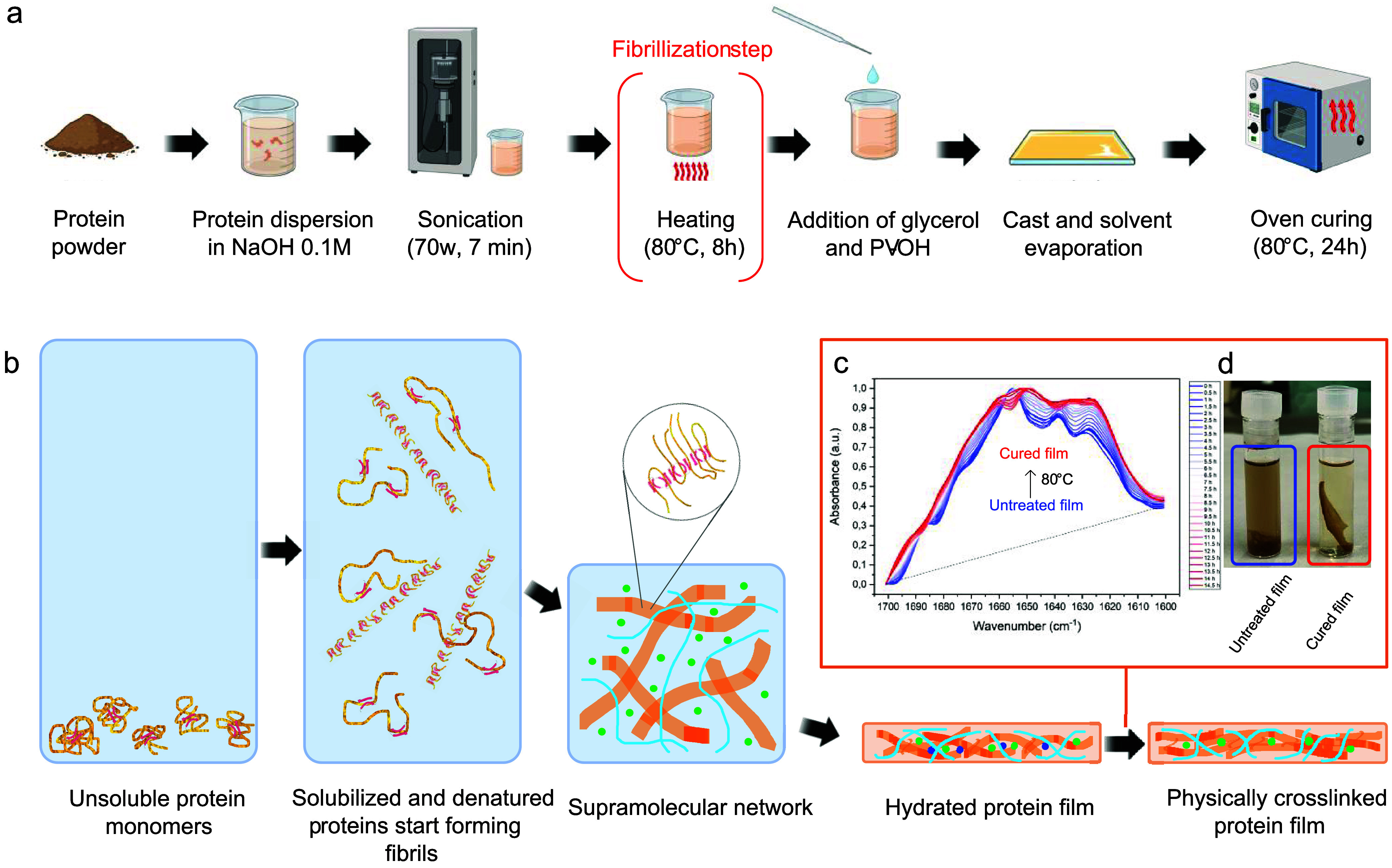
Process and mechanisms underlying the production of blended (BSF protein/PVOH) films. (a) Schematic pathway for the fabrication of the presented BSF protein-based bioplastics. The fibrillization step is highlighted in red brackets [Copyright: Galimberti, M. – 2024, BioRender.com]. (b) Proposed step-by-step mechanisms underlying the formation of the presented BSF protein-based bioplastic. (c) ATR-FTIR absorbance spectra of the amide I (1700–1600 cm^–1^) and amide II (1600–1500 cm^–1^) regions for a film exposed to heating at 80 °C (from 0 to 14.5 h). Heating was performed on a thermostated Ge ATR crystal. (d) Water stability of the BSF film before (left) and after (right) 24 h heating at 80 °C.

Attempts to fabricate free-standing composite films implementing the fibrillization step under acidic conditions (HCl/CH_3_COOH) yielded gels or discontinuous films and fragments, similarly to what was reported in Figure 3a,b; therefore, film fabrication and testing focused on the NaOH route only. Glycerol was used as a plasticizer. On the other hand, being recognized as a food coating material due to its Generally Recognized As Safe (GRAS) status and biodegradability,^79^ PVOH was used to enhance the mechanical performance and the thermal processing of the resulting composite films when compared to previous films constituted solely by BSF proteins.^80^ In this proof-of-concept study, the glycerol content varied between 0 and 50% w/w on the polymer matrix weight (i.e., BSF proteins +PVOH), while PVOH was incorporated at a 1:1 weight ratio on BSF proteins. This ratio was chosen to demonstrate the feasibility of the composite material rather than to define an optimized formulation. Future work is required to investigate the effect of varying the BSF­(af)/PVOH ratio on key performance parameters, such as mechanical, thermal, and barrier properties. For the benefit of the reader, the formulations of prepared films are reported in Table S.2. The structure of the material was expected to be an interpenetrating network of PVOH and BSF protein components, the latter organized in fibrils and amorphous structures with glycerol molecules dispersed within the polymeric network, as outlined in Figure 5b.

A final thermal annealing (oven heating, *T* = 80 °C, 24 h) was carried out on the films to remove the excess water, partially trapped within the polymer chains. Acting as a plasticizer, water could hamper protein–protein interactions, hence leading to materials with lower mechanical properties and water stabilities. The effect of this final thermal treatment was investigated by performing FTIR analysis on a film specimen positioned on a thermostated (80 °C) Ge ATR crystal, monitoring the spectroscopic variation in the amide I region over heating time. As mentioned above, this region is associated with protein conformations, particularly with protein secondary structures.^18,24,62,81,82^ Upon performing the thermal treatment, a decrease in the signal at 1656 cm^–1^ (α-helices) in favor of an increase in the signals at 1645 cm^–1^ (random coils) and 1620 cm^–1^ (intermolecular β-sheets) was observed, as shown in Figure 5c. The relative content of secondary structures was quantified through the second derivative band narrowing (SDBN) method (Figure S.11 and Table S.3), evidencing a significant decrease in the relative content of β-sheets in favor of an increase in intermolecular β-sheets. These results indicate a sensible conformational change of the protein network toward more hydrophobic structures (e.g., intermolecular β-sheets), which can account for the increased water stability of the final films (Figure 5d).

The obtained free-standing bioplastic films were translucent, yellowish, and flexible (Figure 6a). Preliminary biodegradation tests further confirmed the biodegradability of the composite films: BSF­(af)/PVOH/glycerol samples showed visible degradation after 5 days when immersed in a 1% pepsin–water solution at pH 4, whereas no appreciable changes were observed in the reference films exposed to a water solution at the same pH (Figure S.12). Thanks to the presence of PVOH, films could be fused together when melted and pressed at a high temperature, giving mechanically robust products. This result shows the potential of the films prepared in this work to be structured into three-dimensional shapes as shopping bags or other types of packaging materials (Figure 6b).

**Figure 6:**
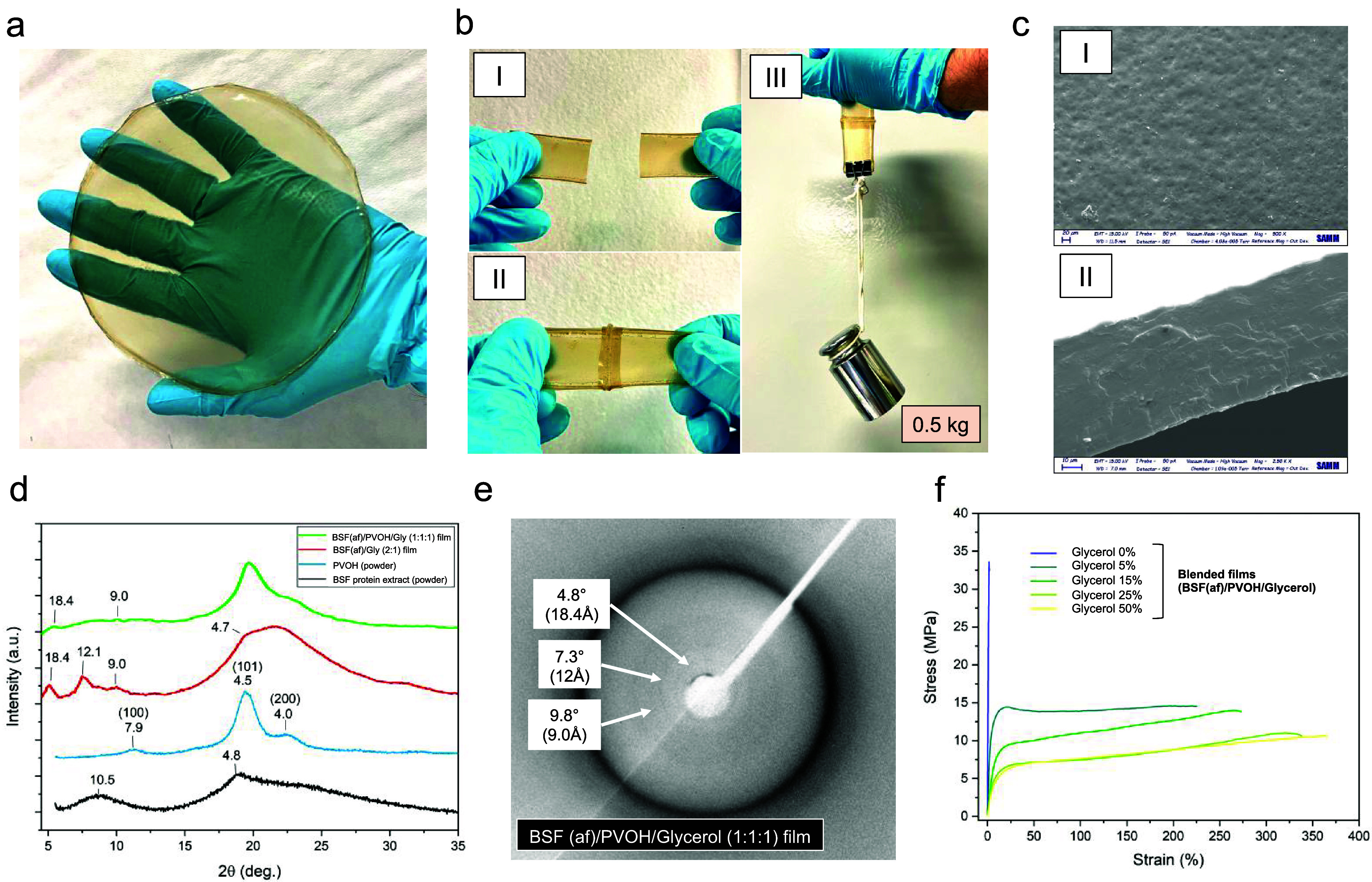
Characterization of blended (BSF­(af)/PVOH/Glycerol) films. (a) Visual appearance of the obtained BSF protein-based bioplastic film. (b) (I) Pieces of bioplastic, unmelted; (II) the two pieces fused together after thermal welding; (III) the resulting fused material under a weight of 0.5 kg, in the direction perpendicular to the melting line. (c) SEM micrographs of the (I) film top surface (scale-bar 20 μm) and (II) section (scale-bar 10 μm). (d) XRD powder patterns of BSF protein extract (black) and PVOH (light blue), bidimensional XRD profiles of unblended film (BSF­(af)/glycerol, 2:1) (red) and blended film (BSF­(af)/PVOH/glycerol, 1:1:1) (green), and (e) bidimensional XRD image of blended film (BSF­(af)/PVOH/glycerol, 1:1:1). (f) Stress–strain curves of blended films (BSF­(af)/PVOH/glycerol) with different contents of glycerol (from 0 to 50% w/w, on matrix weight).

Inspection of the top surfaces of BSF protein bioplastics was performed via scanning electron microscopy (SEM) analysis. The micrographs shown in Figure 6c reveal a homogeneous microstructure of both film’s surface (Figure 6c, I) and section (Figure 6c, II). On the contrary, aggregates of microscopic dimensions (approximately 100 μm) were detected in films prepared by avoiding the fibrillization step (Figure S.13). Reflections due to the crystalline order of fibrils were hardly visible in the XRD profiles of the blended film (Figure 6d). Only in the bidimensional XRD pattern, narrow reflections with very low intensity were noticed at 4.8° (18.4 Å), 7.3° (12.1 Å), and 9.8° (9.0 Å) (Figure 6e). The very low intensity of signals associated with amyloid fibrils could be explained with their lower relative content in this type of composite film due to the addition of a substantial quantitative of PVOH (1:1, on BSF protein weight).

Results from quasi-static tensile tests are seen in Figure 6f. Mechanical parameters were collected and graphed as in Figure S.14. Glycerol was found to have a remarkable effect on the mechanical behavior of the material. The Young’s modulus (E) decreased (from 2236 to 87 MPa) as the glycerol content increased from 0 to 50%, and the ε_b_ increased significantly (from 2.3 to 334%) while σ_b_ decreased (from 34.3 to 9.8 MPa). The mechanical properties of 50% glycerol films were very similar to those of 25% glycerol films, suggesting a plateau effect associated with the glycerol content. Notably, appreciable flexibility was maintained even without glycerol addition, thanks to the presence of PVOH. These findings suggest that tailoring of the material’s mechanical properties, as a function of functional requirements, is feasible by tuning PVOH and glycerol content.

As in previous studies,^21^ films comprising BSF amyloid fibrils (BSF­(af)) showed appreciably higher mechanical performances when compared to films obtained, avoiding the fibrillization step (Figure S.15a,b). In particular, when glycerol was absent (0% w/w), BSF­(af) films displayed higher Young’s modulus (E) (2236 vs 1393 MPa) and stress at break (σ_b_) (34 vs 23 MPa). Besides, films at 50% w/w glycerol content showed a higher deformation at break (ε_b_) (335 vs 239%) while maintaining similar σ_b_ and E. Interestingly, these results are reversed compared to those obtained for films without PVOH, where fibrillization of BSF proteins was found to have a negative effect on mechanical properties (Figure S.15c). This finding could be explained by assuming that BSF amyloid fibrils act as fillers in a composite material, providing tunable mechanical properties as a function of their relative content. Further investigations are needed to validate this hypothesis.

The O_2_ and CO_2_ barrier properties of blended (BSF/PVOH/glycerol, 1:1:1) films were ultimately tested under standard conditions (ASTM D3985:23 °C, RH 0%), while water vapor permeability (WVP) was measured under controlled conditions (38 °C, RH 90%) following the ASTM F 1249 standard. These properties are crucial in applications such as food packaging and medical supplies, as they significantly affect product quality and shelf life.^83–85^

Remarkable low permeability coefficients were found against both O_2_ and CO_2_: 0.0012 and 0.0052 cm^3^·μm·m^–2^·d^–1^·Pa^–1^, respectively, for a nonfibrillizated film, and 0.0008 and 0.0017 cm^3^·μm·m^–2^·d^–1^·Pa^–1^, respectively, for a fibrillizated film (Table S.4). The reduced permeability (P) of the fibrillizated BSF film against both O_2_ and CO_2_ can be primarily ascribed to a lower diffusivity (D) of gas molecules through its polymeric network, which is consistent with the increased structural homogeneity of these films (see Figure S.13). However, it must be considered that the enhanced assembly of polypeptide chains into fibrillar structures concurrently leads to an increase in the apolar character of the matrix, facilitating a higher solubility (S) of O_2_ and CO_2_ within the polymer phase. Given the fundamental relationship governing gas transport in polymers, i.e., *P =* D × S, such an increase in solubility could counterbalance the decrease in permeability due to lower diffusivity. For similar reasons, the higher permeability to CO_2_ can be attributed to the chemical nature of this molecule. Despite being larger in size (thereby displaying lower diffusivity when compared to O_2_), CO_2_ is characterized by a quadrupole moment, which makes it more likely to interact with the polar regions of the protein matrix, thereby increasing its solubility and, hence, its permeability.^86^

To further assess the performance of the fibrillizated BSF film in packaging applications, WVP was measured under the above-mentioned conditions (i.e., 38 °C and 90% RH), displaying a permeability coefficient of 379 g·mm·m^–2^·day^–1^·Pa^–1^. Notably, this number falls to 37 g·mm·m^–2^·day^–1^·Pa^–1^ when an RH of 45% is applied through the test.

### Comparison with Other Materials

Young modulus, tensile strength, and elongation at break of the films based on BSF (af), PVOH, and glycerol were compared with the same properties of different classes of materials (thermoplastics, elastomers, fibers, ceramics, metals, glasses, and natural materials), whose values were taken from the Ansys Granta EduPack database (Figure 7a). Focus is on the comparison with synthetic polymers (i.e., the blue cloud), either oil-based or biobased.

**Figure 7:**
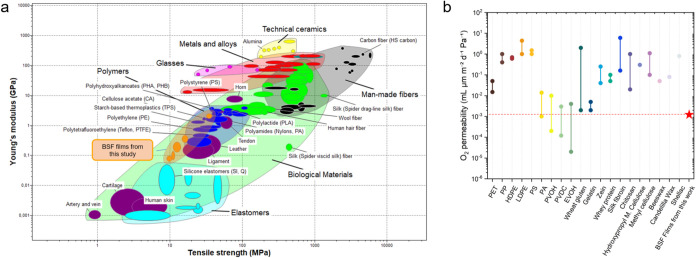
Benchmark to current engineered and natural materials. (a) Comparison of mechanical properties (tensile strength (MPa) vs Young modulus (MPa)) for different synthetic and natural materials. BSF protein-based films presented in this study, characterized by different glycerol loadings (from 0 to 50 wt %), are reported in the orange cloud. Graph generated with the Ansys Granta EduPack 2021 software; (b) O_2_ permeability values of current packaging materials for oil- and biobased polymers. The O_2_ permeability value of the BSF protein-based film presented in this study is reported with a red star. Cumulative data are collected in Table S.5.

The mechanical performance of the films produced in this work lies between that of thermoplastic materials widely used in packaging, such as PE and TPS, and that of biological materials, such as tendons and leather (Figure 7a). In terms of Young’s modulus and tensile strength, the films with 0% w/w glycerol content were in the middle to lower range of plastics, with values comparable to or slightly better than PE, TPS, and PTFE. Tensile strength did not reach the levels of PLA, PHAs, or PS. Deformation at break varied significantly as a function of glycerol content and was lower compared to conventional plastics but higher compared to TPS, PS, and PLA (Figure S.16). On the basis of this comparison, BSF plastics appear to be suitable for applications where moderate mechanical strength and stiffness are sufficient, such as packaging or other disposable products.

In this regard, the O_2_ barrier properties of the bioplastic films of this work were compared with those of films made from either oil-based or biobased polymers. Available O_2_ permeability values are collected in Table S.5. The films in this work showed very low O_2_ permeability values (Figure 7b). They were comparable to those of polyamides, PVOH, PVDC, and EVOH, considered today as the best-performing barrier polymers for packaging applications. The obtained WVP values are of a similar magnitude to those reported for other protein-based bioplastics (526 g·mm·m^–2^·day^–1^·Pa^–1^), but 1 order of magnitude lower than cellulose-based films (2305 g·mm·m^–2^·day^–1^·Pa^–1^).^85^ They are higher than those of high-barrier synthetic polymers, such as PVDC and EVOH (0.014 and 71 g·mm·m^–2^·day^–1^·Pa^–1^, respectively).^85^ These findings suggest that the BSF-based films could be suitable as gas barrier layers within multilayered packaging systems with outer hydrophobic coatings, or for short-shelf life and fully biodegradable packaging.^79,83^

## Conclusions

This work reports, for the first time, the preparation of amyloid fibrils from BSF protein extract. A prolonged thermal treatment (80 °C, *t* > 6 h) was applied to protein water solutions (*C*_protein_ = 5% w/v) at basic pH (= 11.5). This was found as the optimal condition for BSF protein fibrillization due to the higher solubility and surface charge of the protein components. The process appeared to reduce the molecular mass of the protein species: this is thought to be the basis of the high crystalline order detected in mature amyloid fibrils.

Fibrillizated proteins were mixed in water with PVOH and glycerol, and nanostructured bioplastic films were obtained by casting, without using catalysts or cross-linkers. The composite films appeared transparent, flexible, and with mechanical and gas barrier properties comparable to those of oil-based and biobased materials traditionally used for packaging. Fibrils promoted the mechanical reinforcement, while the combination of BSF proteins with PVOH enabled films’ thermal weldability, leading to mechanically robust materials. In this regard, our results indicate that BSF amyloid fibrils combined with 50 wt % of PVOH yield bioplastics whose mechanical and gas barrier properties are comparable to those of current packaging materials. The amount of PVOH, a biodegradable yet oil-derived polymer, can thus be limited to half of the composite material. Given their high aspect ratio, stiffness, and extensive hydrogen-bonding capability, BSF amyloid fibrils could also act as reinforcing nanofillers at low concentrations in PVOH-based bioplastics. This is an aspect that warrants further study.

The biodegradable nature of the bioplastic was confirmed by preliminary biodegradability tests, which showed enzymatic degradation and significant mass loss of the films, thus helping to alleviate the global problems of plastic accumulation and environmental pollution. Additionally, by exploiting the protein fraction of the BSF, this new class of bioplastics may contribute to boosting emerging circular economy models based on the bioconversion of the OFMSW, with its subsequent valorization. The results reported here pave the way for the creation of a new generation of functional and sustainable materials from BSF proteins.

## Experimental Section

### Materials

The proteins utilized in this study were obtained from BSF pupae reared on a substrate mimicking the OFMSW, whose composition was reported in a recent publication^40^ (see modified s-OFMSW). Insects’ rearing and protein extraction followed procedures described in a previous publication.^41^ PVOH (*M*_w_ 146,000–186,000, 99% hydrolyzed) was from Merck/Sigma-Aldrich. Unless otherwise specified, all of the remaining reagents were from Merck/Sigma-Aldrich and have been used without further purification.

### Characterization of Raw BSF Protein Extracts and Solutions

#### Proteomic Analysis

Dried protein extracts were resuspended in a water solution of 0.1 M NaOH, up to a final concentration of 0.5% w/v. A 15 μL portion of the protein extract solution was mixed with an equal volume of Laemmli buffer 4× (comprising 62.5 mM Tris–HCl, pH 6.8, 2% SDS, 25% glycerol, and 0.01% bromophenol blue, BioRad). 20 μL of the mixed solution were loaded onto the SDS-PAGE gel. Gel preparation and electrophoresis run followed established protocols described in previous studies.^87^

Protein identification was achieved by nLC-MS/MS analyses. The SDS-PAGE gels were processed following established protocols described in previous publications.^41,88^ Trypsin (ThermoFisher Scientific) was employed as a digestive enzyme. 8 μL of tryptic-digested samples were injected into a nanochromatography system (UltiMate 3000 RSLCnano System, Thermo Scientific), coupled with a mass spectrometer (LTQ XL, Thermo Scientific). Mass spectrometry data were analyzed using the Mascot search engine (Version 2.3.01) integrated with Proteome Discoverer software (Version 1.2.0 Thermo), referencing the UniProtKB/SwissProt protein database (Uniprot_InsectaRev Insecta_Reviewed/Current/InsectaReviewed), which includes 10,974 sequences and 5,363,805 residues.

#### Solubility Measurements

To determine protein solubility, BSF protein extracts were dissolved in aqueous solutions (0.1% w/v) at varying pH. The pH was adjusted by adding drops of 1 M NaOH or 1 M HCl aqueous solutions and finely tuned with CH_3_COOH or NaHCO_3_ aqueous solutions. Suspensions were homogenized by means of an IKA MS3 agitator (3000 rpm, 10 min) and then centrifuged at 13,400 rpm for 10 min. The soluble protein concentration in the supernatant was quantified through the bicinchoninic acid assay (BCA) (Pierce BCA Protein Assay kit, ThermoFisher Scientific): 100 μL of the BSF protein extract solution were mixed with 2 mL of working reagent in a 2 mL test tube. After gentle agitation, the test tube was incubated at 37 °C for 30 min. Absorbance readings were taken at 562 nm. Protein solubility was determined as the percentage (% w/w) of the protein content in the supernatant on the total extract weight. All measurements were carried out in triplicate for each condition.

To determine whole protein extract solubility, desired suspensions were prepared at a concentration of 5% w/v, transferred to conical tubes, and centrifuged at 13,400 rpm for 10 min. Whole extract solubility was determined gravimetrically by weighing the mass of the supernatant after the solvent was evaporated. All measurements were carried out in triplicate for each condition.

#### Turbidity Measurements

Suspension turbidity was quantified by measuring the sample absorbance at 600 nm (OD_600_) through ultraviolet–visible (UV–vis) spectroscopy. Desired suspensions were prepared at 5% (w/v) and next diluted and tested at a concentration of 0.02% (w/v) to avoid signal saturation phenomena from the spectroscopic measurements. Suspensions were dispensed in PMMA cuvettes (1 cm path length) and analyzed by means of a HP 8452A Diode Array Spectrophotometer, Agilent Technologies. All measurements were carried out in triplicate for each condition.

#### Particle Size and ζ-Potential

Hydrodynamic diameter (*d*_h_) was analyzed through dynamic light scattering (DLS) by means of a Zetasizer Nano (Malvern Instrument). ζ-Potential was analyzed through electrophoretic light scattering (ELS) by means of the same instrument. Desired suspensions were prepared at 5% w/v and then diluted and tested at a concentration of 0.1% w/v.

For DLS measurements, 700 μL of sample were dispensed and analyzed in PMMA cuvettes. Each measurement was collected as the result of 10 runs of 10 s each. For ELS, 700 μL of sample were dispensed and analyzed in a DTS0012 type PMMA cuvette equipped with a Zetasizer Dip Cell. All measurements were carried out in triplicate for each condition.

#### ThT Fluorescence Assay

BSF pupa protein fibrillization was assessed by using ThT fluorescence spectroscopy. Initially, a 2 mM ThT stock solution was prepared by dissolving ThT powder in a Milli-Q water. The resulting suspension was then filtered by using a 0.22 μm syringe filter to remove insoluble particles. A ThT working solution (20 μM) was subsequently prepared by dilution of the ThT stock solution 100-fold in Milli-Q water.

For aggregation experiments, 10 μL of BSF protein suspensions (5% w/v) at desired time steps were combined with 190 μL of the ThT working solution in a 96-well microplate. The ThT fluorescence intensity of the samples was measured immediately after using a Synergy H1 reader (BioTek, Winooski, VT) with excitation at 450 nm and emission at 490 nm, maintaining a constant temperature of 25 °C and the same instrument gain over all of the experiments. All measurements were carried out in triplicate for each condition.

#### SDS-PAGE on Fibrillizating Solutions

The MW profile evolution under prolonged heating at 80 °C was monitored through SDS-PAGE. Desired suspensions were prepared at 5% w/v and exposed to 80 °C in a thermomixer. At the desired time step, aliquots were withdrawn from the suspensions and diluted to 0.5% (w/v) in the same buffers. A 15 μL aliquot of this suspension was mixed with 5 μL of 4× Laemmli buffer (comprising 62.5 mM Tris–HCl, pH 6.8, 2% SDS, 25% glycerol, and 0.01% bromophenol blue, BioRad), flash-freeze in liquid N_2_, and stored at −80 °C until used (max. 24 h). The 20 μL of the mixed solution were loaded onto the SDS-PAGE gel. Gel preparation and electrophoresis run followed established protocols described in previous studies.^87^

#### TEM Microscopy

Morphological structures of BSF proteins were monitored by TEM analyses. Solubilized BSF protein liquid samples (5% w/v) at desired time steps were first diluted in Milli-Q water to 0.1% w/v and then applied (6 μL) to a carbon-coated 200-mesh copper grid and left for 3 min. Excess solution on the grids was absorbed with filter paper. After being air-dried (3 days), grids were examined with a Philips CM200 (Agilent Technologies) electron microscope working at an accelerating voltage of 120 kV.

### Fabrication and Characterization of Bioplastics

#### Fabrication of BSF Bioplastic Films from NaOH, HCl, and CH_3_COOH Solutions

250 mg of BSF protein were poured into a 10 mL becher, and 5 mL of a 0.1 M NaOH, 0.1 M HCl, or 5 M CH_3_COOH Milli-Q water solution were added to the proteins. The mixture was stirred for 5 min to obtain a homogeneous suspension. The suspension was next probe sonicated (Branson SFX550, 13% amplitude (70W), 7s ON + 3s OFF, 7 min total ON) and centrifuged (9000 rpm, 15 min, 20 °C). The protein suspension was poured into a new 10 mL beaker, and glycerol was added (50% on extract weight). The whole suspension was stirred for an additional 10 min. The suspension was casted in a PDMS mold (25 mm × 50 mm) and left to dry at R.T. for 72 h.

#### Production of Amyloid Fibrils

250 mg of BSF protein were poured into a 10 mL becher. 4.5 mL of Milli-Q water were added to proteins, followed by 0.5 mL of 1 M NaOH. The mixture was stirred for 5 min to obtain a homogeneous suspension. The suspension was next probe sonicated (Branson SFX550, 13% amplitude (70W), 7s ON + 3s OFF, 7 min total ON) and centrifuged (9000 rpm, 15 min, 20 °C). The obtained solution was then heated in a closed double-neck flask with a reflux condenser mounted on it (magnetic stirring 300 rpm, 80 °C in oil bath, 0–24 h).

#### Fabrication of BSF Protein/Glycerol (2:1) Films

For fibrillizated films, amyloid fibrils were produced as described above (see *Production of amyloid fibrils*). After 8 h of heating, the protein solution was poured into a new 10 mL beaker, and glycerol (50% w/w, on BSF protein weight) was added. The whole solution was stirred for 1 h, cast in a PDMS mold (25 × 50), and left to dry at R.T. for 72 h. The obtained films were eventually submitted to a heat treatment in an oven at 80 °C for 24 h. Unless otherwise specified, films were stored in plastic bags in the dark until use. Reference films (nonfibrillizated) followed the same procedure, avoiding the fibrillization step (80 °C, 8 h).

#### Fabrication of Blended BSF Protein/PVOH/Glycerol (1:1:1) Films

For fibrillizated films, amyloid fibrils were produced as described above (see *Production of amyloid fibrils)*. In parallel, a 5% w/v PV–OH water solution was prepared by dissolving PV–OH in Milli-Q water at 95 °C (oil bath) under vigorous stirring (1000 rpm) for 8 h. After 8 h of heating, the protein suspension was poured into a new 10 mL beaker, and glycerol (50% on extract weight) and PV–OH (100% on extract weight) were added. The whole suspension was stirred for 10 additional minutes, cast in a PDMS mold (25 mm × 50 mm), and left to dry at R.T. for 72 h. The obtained bioplastic was eventually submitted to a heat treatment in an oven at 80 °C for 24 h. Reference films (nonfibrillizated) followed the same procedure, avoiding the fibrillization step (80 °C, 8 h).

#### XRD Measurements

XRD patterns were obtained by a Bruker D2 automatic diffractometer with nickel-filtered Cu Kα radiation, operated at a step size of 0.03° with 164 s/step. Two-dimensional wide-angle X-ray diffraction (2D-WAXD) patterns were obtained by a D8 QUEST Bruker diffractometer (Cu Kα radiation) by sending the X-ray beam parallel (EDGE patterns) or perpendicular (THROUGH patterns) to the film surface.

The characteristic interplanar spacings (*d*) were calculated from Bragg’s equation:

nλ=2dsenθ

where *n* is the order of diffraction (an integer, usually 1), λ is the wavelength of the incident X-ray, *d* is the interplanar spacing of the crystal planes, and θ is the angle of incidence (Bragg angle).

#### ATR-FTIR Measurements

ATR-FTIR measurements on bioplastic films were carried out using a Nicolet iS5 with KBr windows (Thermo Scientific) imaging system equipped with a VeeMAX II ATR (Pyke technologies) with a germanium (Ge) crystal under nitrogen (N_2_) flux. The IR absorption spectra were recorded in the region of 600–4000 cm^–1^ based on 64 scans and a resolution of 8 cm^–1^. When used to mimic the oven drying step, the Ge crystal was heated at 80 °C, recording spectra every 30 min. Background subtraction was performed before each measurement.

For secondary structure evaluation and relative quantification, the second derivative band narrowing method (SDBN) was used. Raw IR spectra in the amide I region (1700–1600 cm^–1^) were extrapolated and baseline corrected. The second derivative function was next obtained. Data fitting was performed on peaks above zero for the negative second derivative function using the Fit Peaks PRO algorithm. Relative quantification of secondary structures was due by calculating the area under each peak as a percentage of the total peak areas. The secondary structure assignation was from the literature.^18,24,62,81,82^

#### SEM Microscopy

Films’ specimens were mounted on aluminum stubs using conductive carbon tape, sputter-coated with pure gold (S150B Edwards), and observed under an extended pressure SEM EVO 50 EP (Zeiss) equipped with a X-ray spectrometer EDS Quantax 200 6/30 (Bruker). Observations were performed under high-vacuum conditions at 15 kV.

#### Gas Permeability Tests

Gas barrier properties were evaluated through the O_2_ and CO_2_ permeability tests. Round-shaped, 140 mm diameter films were prepared for the test by opportunely scaling ingredients’ quantities to give specimens of approximately 80 μm thick. Films were conditioned for 7 days at 23 °C, 0% RH in a Totalperm CarHum instrument (ExtraSolution, PermTech, Italy), and then submitted to the test under the same conditions (23 °C, 0% RH, 100% partial pressure gas test). The sample surface was 50 cm^2^, and the barometric pressure was 1 bar. Permeability (P) values were obtained from transmission rate (TR) values as from the following equation:

$$P=\frac{\mathrm{TR}}{\Delta P}\times t$$

where *t* is the sample’s thickness (≈80 μm), and Δ*P* is the differential pressure across the two sides of the specimen (1 bar). Water vapor permeability (WVP) of the BSF protein-based films was determined by using the same instrument (Totalperm CarHum instrument (ExtraSolution, PermTech, Italy)), equipped with a surface reducer cell to accommodate film specimens to reach an exposed area of 2.01 cm^2^. Measurements were performed at 38 °C under a controlled relative humidity gradient (carrier side: 0% RH; test side: 90% RH), with a carrier gas flow rate of 71.2 mL·min^–1^ and a differential water vapor partial pressure of 60 mbar across the film. Water vapor transmission rate (WVTR) was obtained directly from the instrument output, and the WVP (g·mm·m^–2^·day^–1^·Pa^–1^) was calculated by normalizing the WVTR to film thickness according to the following equation:

P=TR×t

#### Mechanical Tests

Mechanical properties of bioplastic films were evaluated through quasi-static mechanical tensile tests. Dog bone-shaped specimens (21 mm × 7.2 mm × 0.150 mm) were punched out from cast bioplastic films (*n* = 3 for each formulation). Films were conditioned at 80 °C for 24 h and then to 30 °C and 40% RH for 24 h. Tensile stress–strain curves were obtained under quasi-static conditions using a dynamic mechanical analyzer (MCR702, Anton Paar) equipped with tension clamps. A preload of 0.1 N was applied, and the elongation rate was set at 2 mm min^–1^.

#### Biodegradability Tests

Biodegradability experiments were conducted by exposing films of standardized dimensions (5 × 20 mm^2^) in a 1% w/v water-pepsin solution (pH 4) kept at 36 °C under magnetic stirring (300 rpm), as reported in previous research.^20^ For reference, films were exposed to the same water solution (pH 4 at 36 °C) without pepsin. The degradability of the films was visually examined by monitoring the structure integrity.

## Supplementary Material



## Notes

Experimental details, materials, and methods, including photographs of the experimental setup (E. Testa et al.—amyloid-like self-assembling BSF) (PDF)
