# Supplementary material for: Amyloid-Like Self-Assembling of Black Soldier Fly Proteins and Development of Bioplastics
Source: ACS Sustain Chem Eng. 2025 Oct 18;13(43):18713–26. doi: 10.1021/acssuschemeng.5c07418 (PMC12588267; doi:10.1021/acssuschemeng.5c07418)
Supplement: Supplementary file 1 [file sc5c07418_si_001.pdf]

Supporting Information

Amyloid-like self-assembling of Black Soldier Fly proteins and development of bioplastics

Edoardo Testa,[1] Elisa Fasoli,[1] Paola Rizzo,[2] Morena Casartelli,[3] Gianluca Molla,[4] Gianluca Tettamanti,[4] Maurizio Galimberti [1]*

[1] Politecnico di Milano, Department of Chemistry, Materials and Chemical Engineering "G. Natta", Via Mancinelli 7, 20131 Milano, Italy

[2] Università degli Studi di Salerno, Department of Chemistry and Biology and INSTM Research Unit, Via Giovanni Paolo II 132, University of Salerno, 84084 Fisciano, Italy

[3] Università degli Studi di Milano, Department of Biosciences, Via Celoria 26, 20133 Milano, Italy

[4] Università degli Studi dell'Insubria, Department of Biotechnology and Life Sciences, Via J. H. Dunant 3, 21100 Varese, Italy

*Corresponding author:

Maurizio Galimberti (maurizio.galimberti@polimi.it)

Keywords: Bioplastics, proteins, *Hermetia illucens*, amyloid fibrils, packaging

Contents of Supporting Information

This document contains a total of 17 pages, including 5 tables, 16 figures, 1 supplementary text, and references.

Contents

Supplementary Tables

Table S. 1. Table of proteins identified by nLC-MS/MS analysis. The table reports molecular weight, Mascot score, and number of identified peptides associated with each identification. Results are representative of common identifications between three different biological replicates. Procedure was reported in the experimental section (Proteomic characterization). 5
Table S. 2 Relative composition by weight for the bioplastic films proposed in this study. 11
Table S. 3. Relative content of protein secondary and quaternary structures in BSF protein films exposed to prolonged heating. Quantification performed by the SDBN method. 12
Table S. 4 Gas barrier properties of blended (BSF protein/PVOH) films. The table reports the O_2 (OTR) and CO_2 (CO_2TR) transmission rates and computed permeability coefficients (OP and CO_2P) for fibrillated and non-fibrillated blended films (50% w/w glycerol) determined under standard conditions (ASTM D3985: 23°C, RH 0%). 15
Table S. 5 Oxygen permeability (OP) of main oil-based polymers and natural polymers. 17

Supplementary Figures

Figure S. 1. Schematic process for the extraction of BSF proteins after the bioconversion of OFMSW. 4
Figure S. 2. SDS-PAGE analysis of BSF pupae protein extracts dispersed in Milli-Q water. Column "a" and "b" are representative of two different biological replicates. Procedure for the analysis was reported in the experimental section (Proteomic characterization). 4
Figure S. 3. ζ-potential (mV) (black dots) and soluble protein content (% on extract weight) (grey dots) of BSF protein extracts dispersions at 0.1% w/v as a function of pH. Data are shown as mean ± s.d.; n = 3. 5
Figure S. 4. TEM micrographs of BSF protein extract suspension at 0.1% w/v at its maximum solubility point (NaOH 0.1M, pH=12). Scale bar: 100 nm (left); 50 nm (right). 6
Figure S. 5. Visual appearance of BSF protein extract suspensions (0.5% w/v) before and after the ultrasonication step (Branson SFX550, 13% amplitude, 7s ON + 3s OFF, 7 min total ON) for the three tested solvating conditions (i.e. 0.1M HCl, 5M CH_3COOH and 0.1M NaOH). 6
Figure S. 6 SDS-PAGE profiles (a) of BSF protein extracts dissolved in alkaline environment (0.1M NaOH, pH 11.5) before and after ultrasonication and (b) of BSF protein extracts dissolved in acidic environment (0.1M HCl, pH 2) before and after ultrasonication. 7
Figure S. 7. Particle size and ζ-potential of BSF proteins in 0.1M HCl, 5M CH_3COOH and 0.1M NaOH. Displayed data are representative of BSF protein suspension at 0.1% w/v exposed to the ultrasonication step (Branson SFX550, 13% amplitude, 7s ON + 3s OFF, 7 min total ON). DLS measurements were performed immediately after the process to avoid aggregation phenomena. (a) Particle size distribution (PSD) by volume of BSF protein extract suspensions in 0.1M HCl; (b) PSD by volume of BSF protein extract suspensions in 5M CH_3COOH; (c) PSD by volume of BSF protein extract suspensions in 0.1M NaOH; (d) ζ-potential (mV) values for BSF protein extract suspensions for the three tested solvating conditions. For (a), (b) and (c) graphs are representative of three independent measurements. Insets display the associated correlograms. For (d) data are shown as mean ± s.d.; n = 3. 8
Figure S. 8 Evolution of MW profiles in BSF proteins suspensions exposed to prolonged heating. SDS-PAGE runs for 0.1M NaOH suspensions of BSF proteins exposed to different heating times at 80°C. For each tested sample, aliquots were withdrawn from liquid suspensions (5% w/v) of BSF proteins at the specified time steps, diluted to 0.5% w/v and mixed with Laemmli buffer 4x, flash-freezed into liquid N_2, stored overnight at -80°C and loaded into the gel. 9
Figure S. 9 ThT fluorescence emission (Relative fluorescence unit, RFU) at 490 nm for BSF proteins suspensions in 0.1M NaOH (red) or 0.1M HCl (yellow) exposed to 80°C heating. Data are shown as mean ± s.d.; n = 3. 10
Figure S. 10 Comparison between BSF proteins nanostructuring as a function of heat exposure. In blue box, TEM micrographs of 0.1M NaOH suspensions of BSF protein exposed to room temperature at different time points (4, 8, 24h) after the sonication step. In the red box the same suspensions were submitted to heating at 80°C. Starting samples (5% w/v) were diluted at desired time steps in Milli-Q water to 0.1% w/v and immediately transferred (6 µL) onto the carbon coated copper grid for TEM analysis. 10

Figure S. 11. Effect of prolonged heating at 80°C on the film's protein structure. (a) ATR-FTIR absorbance spectra of the Amide I (1700-1600 cm^{-1}) and Amide II (1600-1500 cm^{-1}) regions for a film exposed to heating at 80°C (from 0 to 14.5 hours). Heating was performed on a thermostated Ge ATR crystal. (b) Inverted second derivative ($-dA^2/d^2\upsilon$) of ATR-FTIR absorbance spectra for the same film at time-zero (blue) and after 14.5 hours (red) heating at 80°C. Secondary structure assignation was from literature. 11
Figure S. 12 Biodegradability of blended films (BSF protein/PVOH/Glycerol, 1:1:1). Visual appearance of the film before and after the degradation tests: (a) pristine film; (b) after 5 days in a 1% pepsin water solution (pH = 4) at 36°C; (c) after 5 days in a water solution (pH = 4) at 36°C, without pepsin. 12
Figure S. 13 SEM micrographs of the top surfaces of blended films (BSF protein/PVOH/Glycerol, 1:1:1) obtained with (I, scale bar: 20μm) and without (II and III, scale bars: 100 and 20 μm, respectively) the fibrillization process. 12
Figure S. 14 Tensile properties of fibrillated blended films at varying loadings of glycerol. (a) Elongation at break (ε_B) values; (b) Stress at break (σ_B) values; (c) Young's Modulus (E) values. Data are shown as mean ± SD (n=3). 13
Figure S. 15 Stress-strain curves of fibrillated vs. non fibrillated films. (a) Blended films (BSF protein/PVOH/Glycerol, 1:1:0), 0% w/w glycerol loading; (b) Blended films (BSF protein/PVOH/Glycerol, 1:1:1), 50% w/w glycerol loading; (c) Films without PVOH (BSF protein/Glycerol, 2:1), 50% w/w glycerol loading. Tests were conducted in triplicate for each condition. The inset table reports the mechanical parameters associated with the graph. Data are reported as mean ± SD (n=3). 15
Figure S. 16. Tensile mechanical properties of blended films - Benchmark to other materials. Elongation (% strain) vs. Tensile strength (MPa). Data and graphs generated with the software Ansys Granta EduPack 2021. 16

Supplementary Texts

Text S.1 Evolution of MW profiles in BSF proteins suspensions exposed to prolonged heating 9

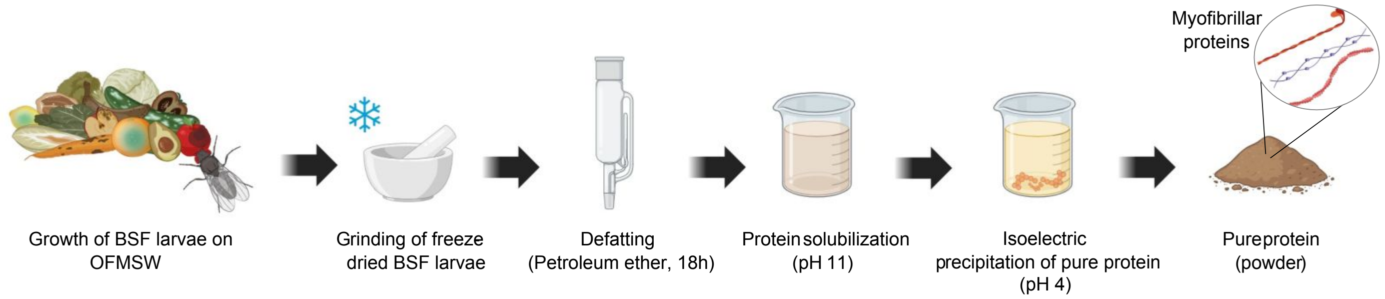

Figure S. 1. Schematic process for the extraction of BSF proteins after the bioconversion of OFMSW.

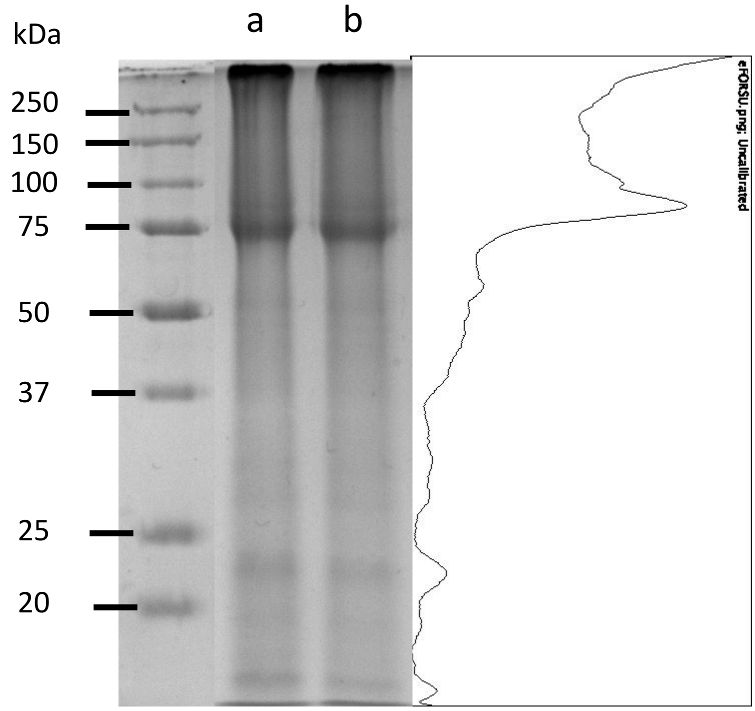

Figure S. 2. SDS-PAGE analysis of BSF pupae protein extracts dispersed in Milli-Q water. Column “a” and “b” are representative of two different biological replicates. Procedure for the analysis was reported in the experimental section (Proteomic characterization).

Table S. 1. Table of proteins identified by nLC-MS/MS analysis. The table reports molecular weight, Mascot score, and number of identified peptides associated with each identification. Results are representative of common identifications between three different biological replicates. Procedure was reported in the experimental section (Proteomic characterization).

Protein name	Accession number	Mr (Da)	Mascot Score	N° peptides
Isoform Embryonic of Tropomyosin-2	P09491-2	32901	1249	11
Muscle-specific protein 20	P14318	20292	336	3
Tropomyosin	P31816	32477	932	12
Troponin I	P36188	30234	147	3
Actin-2, muscle-specific	P45885	42118	640	5
Troponin C, isoform 3	P47949	17742	163	3
ATP synthase subunit beta, mitochondrial	Q05825	54074	447	8
Tropomyosin-2	Q1HPQ0	32823	2107	15
Isoform 3 of Tropomyosin-2	Q1HPQ0-3	32558	1966	15
Tropomyosin-1	Q1HPU0	32603	1390	11
Tropomyosin Lep s 1.0101	Q8T380	32508	137	6

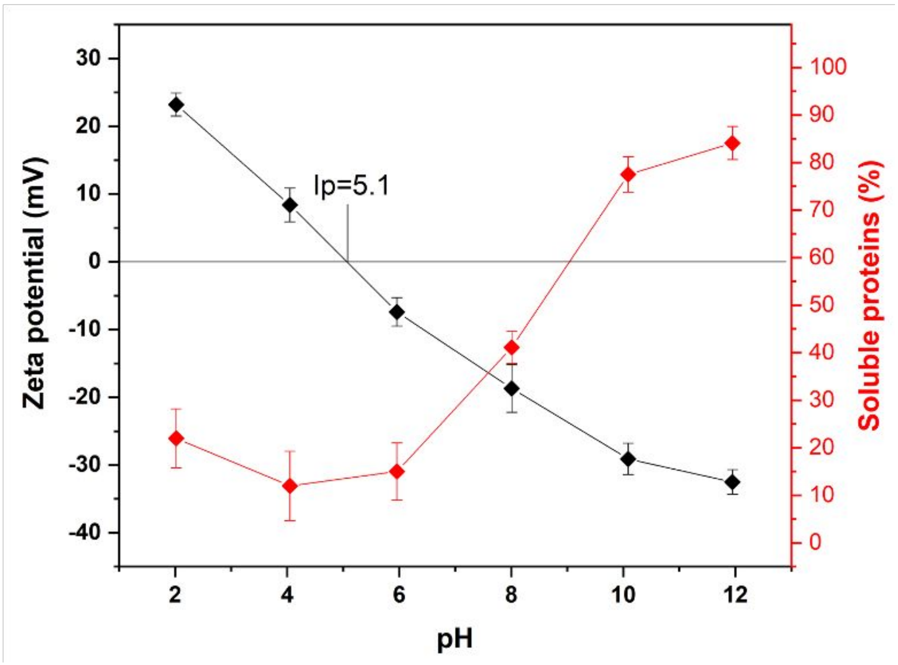

Figure S. 3. ζ-potential (mV) (black dots) and soluble protein content (% on extract weight) (grey dots) of BSF protein extracts dispersions at 0.1% w/v as a function of pH. Data are shown as mean ± s.d.; n = 3.

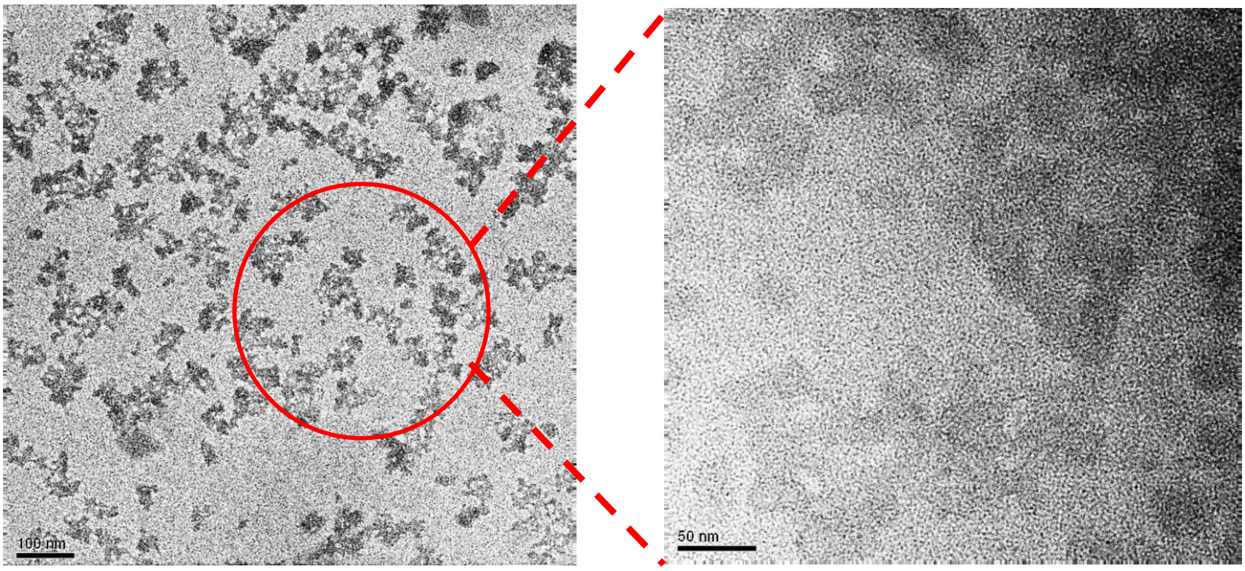

Figure S. 4. TEM micrographs of BSF protein extract suspension at 0.1% w/v at its maximum solubility point (NaOH 0.1M, pH=12). Scale bar: 100 nm (left); 50 nm (right).

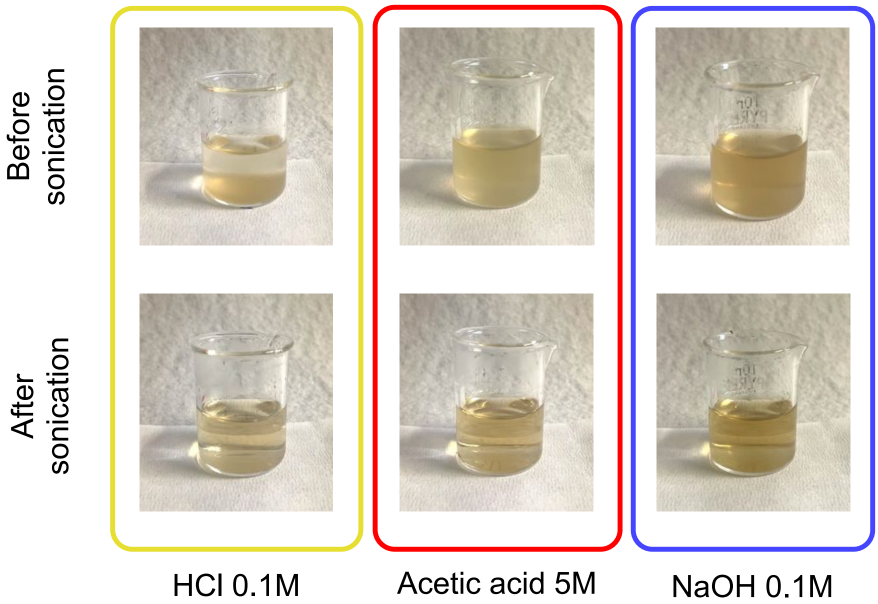

Figure S. 5. Visual appearance of BSF protein extract suspensions (0.5% w/v) before and after the ultrasonication step (Branson SFX550, 13% amplitude, 7s ON + 3s OFF, 7 min total ON) for the three tested solvating conditions (i.e. 0.1M HCl, 5M CH_3COOH and 0.1M NaOH).

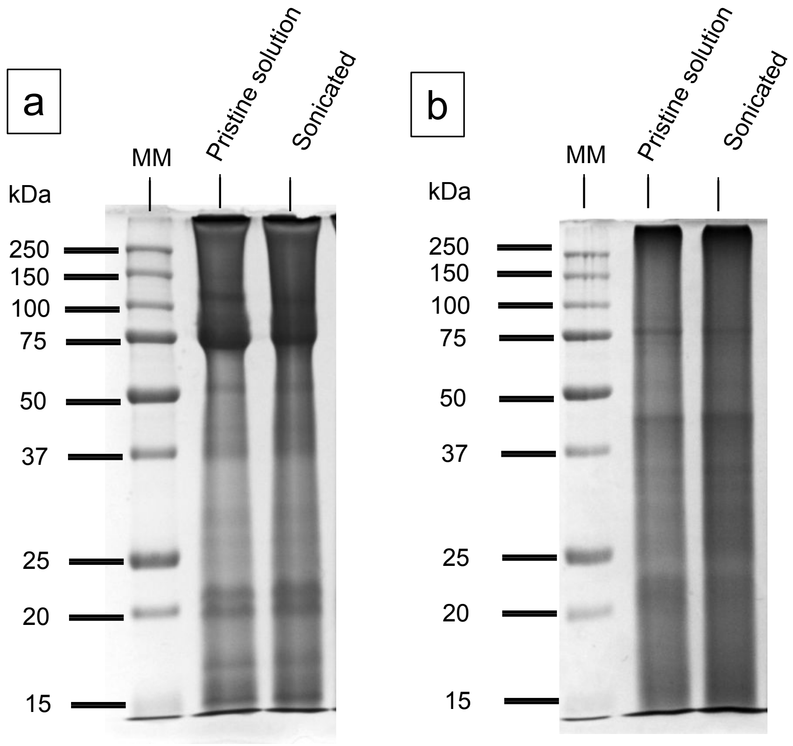

Figure S. 6 SDS-PAGE profiles **(a)** of BSF protein extracts dissolved in alkaline environment (0.1M NaOH, pH 11.5) before and after ultrasonication and **(b)** of BSF protein extracts dissolved in acidic environment (0.1M HCl, pH 2) before and after ultrasonication.

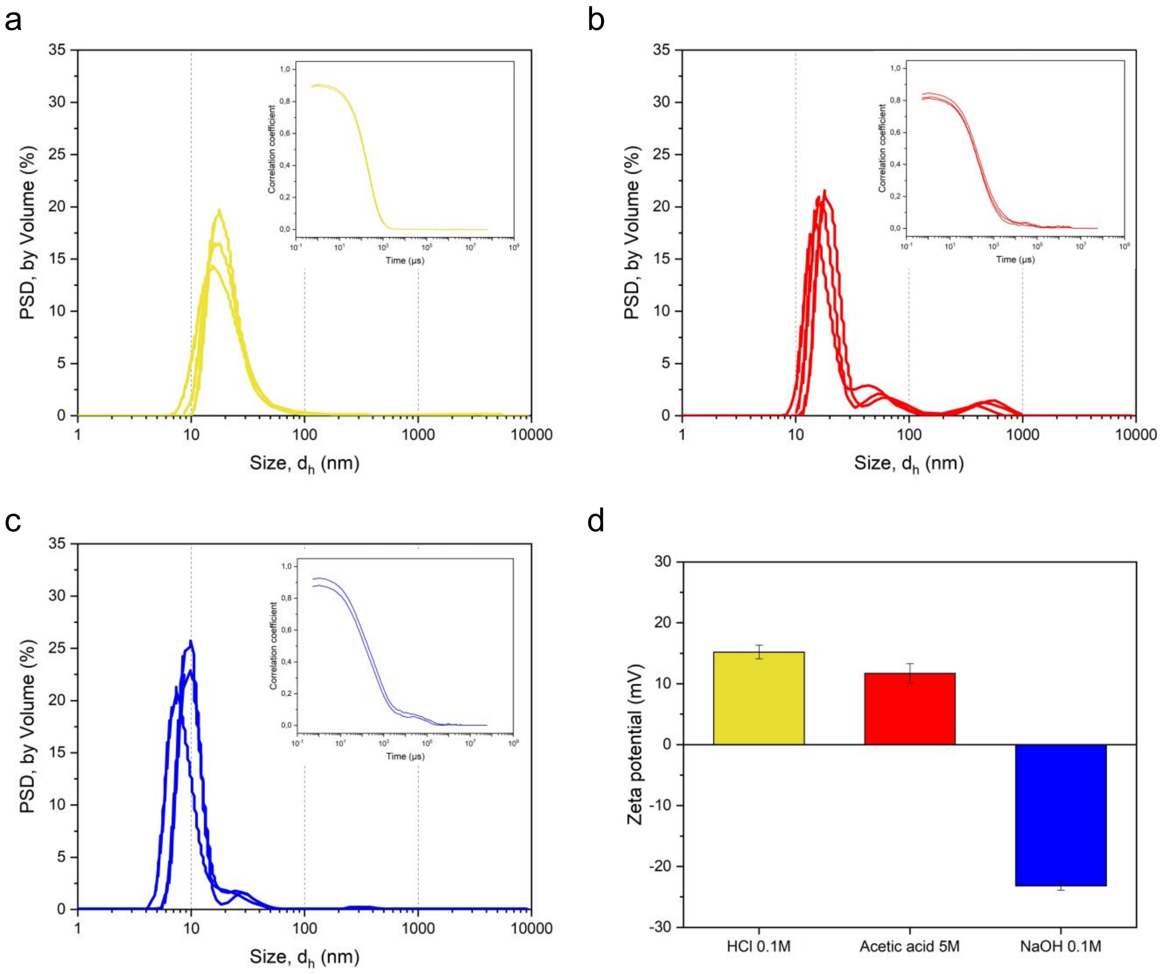

Figure S. 7. Particle size and ζ-potential of BSF proteins in 0.1M HCl, 5M CH_3COOH and 0.1M NaOH. Displayed data are representative of BSF protein suspension at 0.1% w/v exposed to the ultrasonication step (Branson SFX550, 13% amplitude, 7s ON + 3s OFF, 7 min total ON). DLS measurements were performed immediately after the process to avoid aggregation phenomena. **(a)** Particle size distribution (PSD) by volume of BSF protein extract suspensions in 0.1M HCl; **(b)** PSD by volume of BSF protein extract suspensions in 5M CH_3COOH; **(c)** PSD by volume of BSF protein extract suspensions in 0.1M NaOH; **(d)** ζ-potential (mV) values for BSF protein extract suspensions for the three tested solvating conditions. For (a), (b) and (c) graphs are representative of three independent measurements. Insets display the associated correlograms. For (d) data are shown as mean ± s.d.; n = 3.

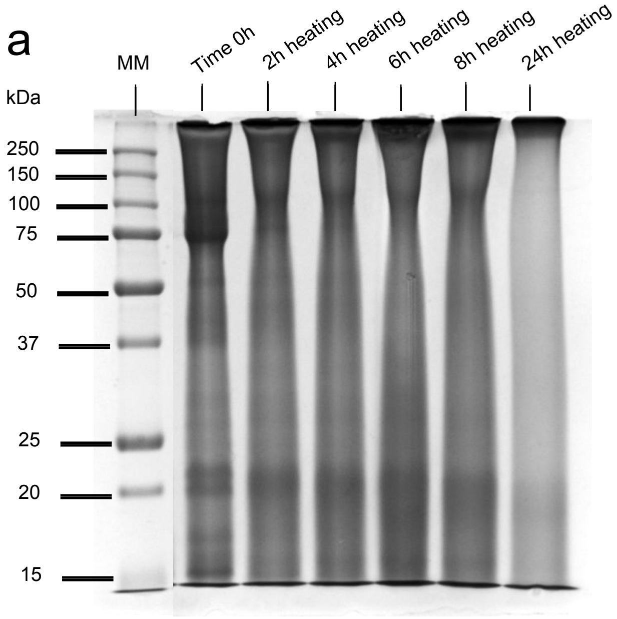

Figure S. 8 Evolution of MW profiles in BSF proteins suspensions exposed to prolonged heating. SDS-PAGE runs for 0.1M NaOH suspensions of BSF proteins exposed to different heating times at 80°C. For each tested sample, aliquots were withdrawn from liquid suspensions (5% w/v) of BSF proteins at the specified time steps, diluted to 0.5% w/v and mixed with Laemmli buffer 4x, flash-freezed into liquid N_2, stored overnight at -80°C and loaded into the gel.

Text S.1 Evolution of MW profiles in BSF proteins suspensions exposed to prolonged heating.

SDS-PAGE runs evidenced, for BSF protein suspensions in 0.1M NaOH, an overall fading of the protein bands as the heating time increased (**Figure S. 8**). It can be evidenced that the predominant band around 75 kDa disappeared after 2h heating, whilst bands in the region between 50-37 kDa and below 20 kDa faded as the heating time increased. Bands around 20 kDa also appeared slightly downgraded. Looking at the top of the gel, darkening of the region above 250 kDa (i.e., material which did not enter the gel) was observed starting from 4h heating. After 24h heating, the gel column appeared almost cleared except for the region above 250 kDa and a low signal below 20 kDa.

Overall, the occurrence of hydrolysis in the upper half of the gel could be hardly evidenced. Here, it may be assumed that material is instead rapidly de-aggregated, as suggested by the disappearance of the band at 75 kDa after only 2h heating. The fading of bands and overall smearing in this region could be hypothesized to be attributed to protein hydrolysis. Hydrolysis is more visible in the lower region of the gel, where shortening of peptides caused an appreciable down-shift of bands towards the bottom. Besides, it could be assumed from the results, that bigger oligomers were formed concurrently as the heating time increased, as visible in the upper part of the gel in the region above 250 kDa.

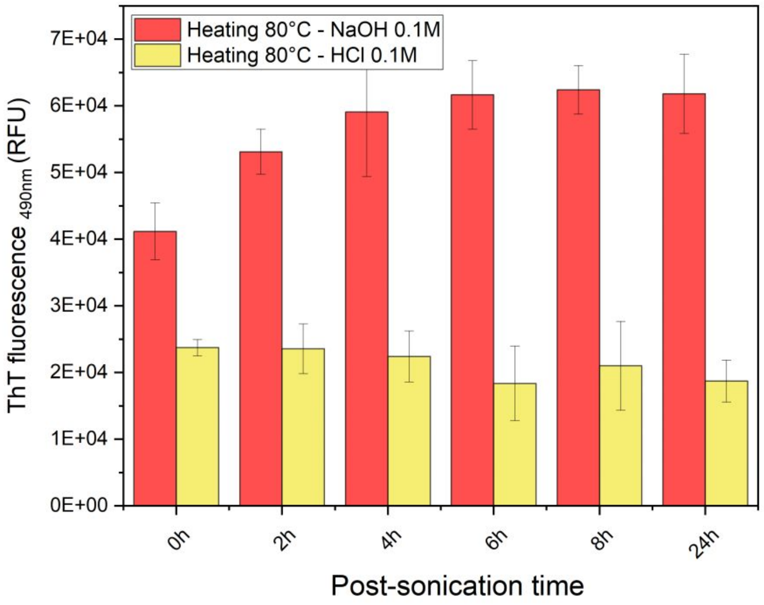

Figure S. 9 ThT fluorescence emission (Relative fluorescence unit, RFU) at 490 nm for BSF proteins suspensions in 0.1M NaOH (red) or 0.1M HCl (yellow) exposed to 80°C heating. Data are shown as mean ± s.d.; n = 3.

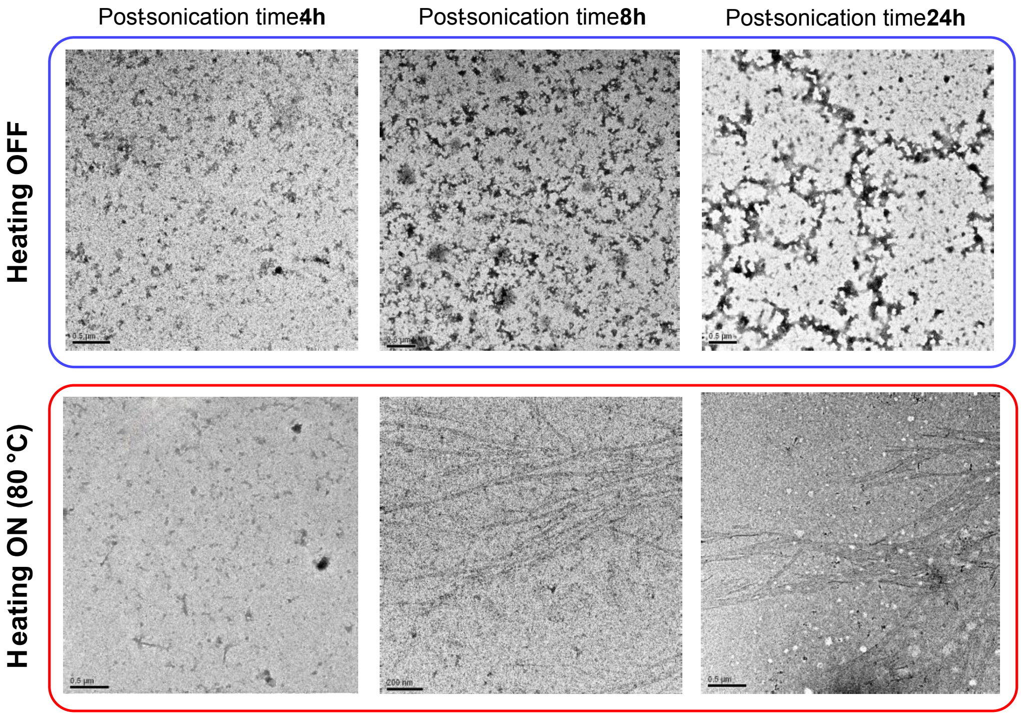

Figure S. 10 Comparison between BSF proteins nanostructuring as a function of heat exposure. In blue box, TEM micrographs of 0.1M NaOH suspensions of BSF protein exposed to room temperature at different time points (4, 8, 24h) after the sonication step. In the red box the same suspensions were submitted to heating at 80°C. Starting samples (5% w/v) were diluted at desired time steps in Milli-Q water to 0.1% w/v and immediately transferred (6 µl) onto the carbon coated copper grid for TEM analysis.

Table S. 2 Relative composition by weight for the bioplastic films proposed in this study.

	Fibrillization step (0.1M NaOH, 8h, 80°C)	Relative contents in final films [%] *		
		BSF protein extract	PVOH	Glycerol
BSF(af)/PVOH/Gly 0% [a]	v	50	50	0
BSF(af)/PVOH/Gly 10%	v	45	45	9
BSF(af)/PVOH/Gly 15%	v	43	43	13
BSF(af)/PVOH/Gly 25%	v	40	40	20
BSF(af)/PVOH/Gly 50%	v	33	33	33
BSF/PVOH/Gly 0%	x	50	50	0
BSF/PVOH/Gly 50%	x	33	33	33

[a] w/w, on matrix weight. Matrix = PVOH + BSF proteins.

* Determined considering complete dehydration of the films.

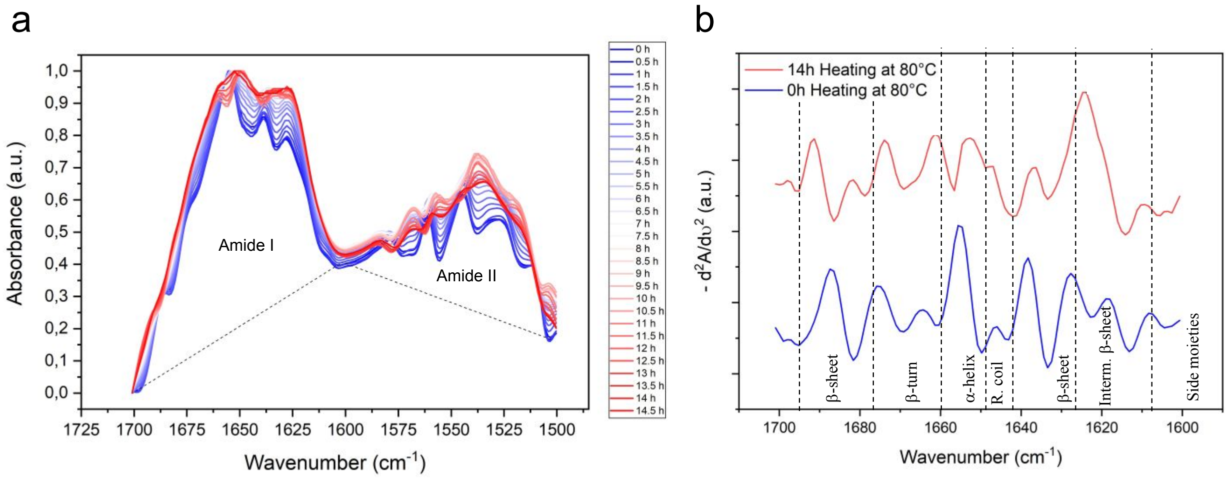

Figure S. 11. Effect of prolonged heating at 80°C on the film's protein structure. (a) ATR-FTIR absorbance spectra of the Amide I (1700-1600 cm^{-1}) and Amide II (1600-1500 cm^{-1}) regions for a film exposed to heating at 80°C (from 0 to 14.5 hours). Heating was performed on a thermostated Ge ATR crystal. (b) Inverted second derivative ($-dA^2/d^2\upsilon$) of ATR-FTIR absorbance spectra for the same film at time-zero (blue) and after 14.5 hours (red) heating at 80°C. Secondary structure assignation was from literature.

Table S. 3. Relative content of protein secondary and quaternary structures in BSF protein films exposed to prolonged heating. Quantification performed by the SDBN method.

	Relative content [%]	
**Secondary structures**	**Untreated film**	**Cured film**
β-sheet	48,4	12,4
β-turn	11,9	26,2
α-helix	33,6	16,5
Random coil	0,0	1,8
Interm. β-sheet	6,1	43,1
Side moieties	1,4	0,0
**Total**	**100,0**	**100,0**

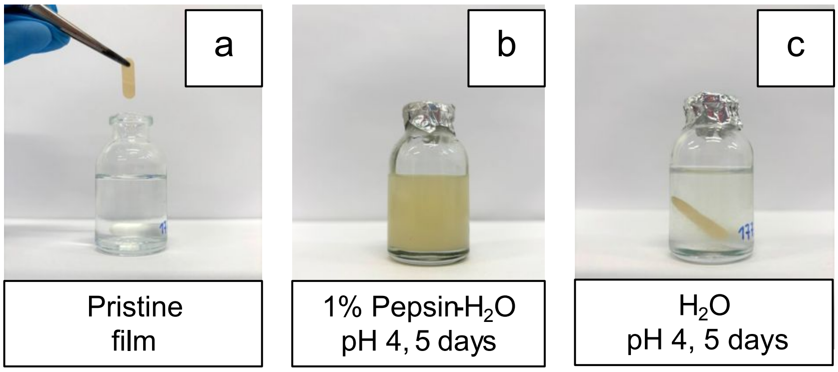

Figure S. 12 Biodegradability of blended films (BSF protein/PVOH/Glycerol, 1:1:1). Visual appearance of the film before and after the degradation tests: (a) pristine film; (b) after 5 days in a 1% pepsin water solution (pH = 4) at 36°C; (c) after 5 days in a water solution (pH = 4) at 36°C, without pepsin.

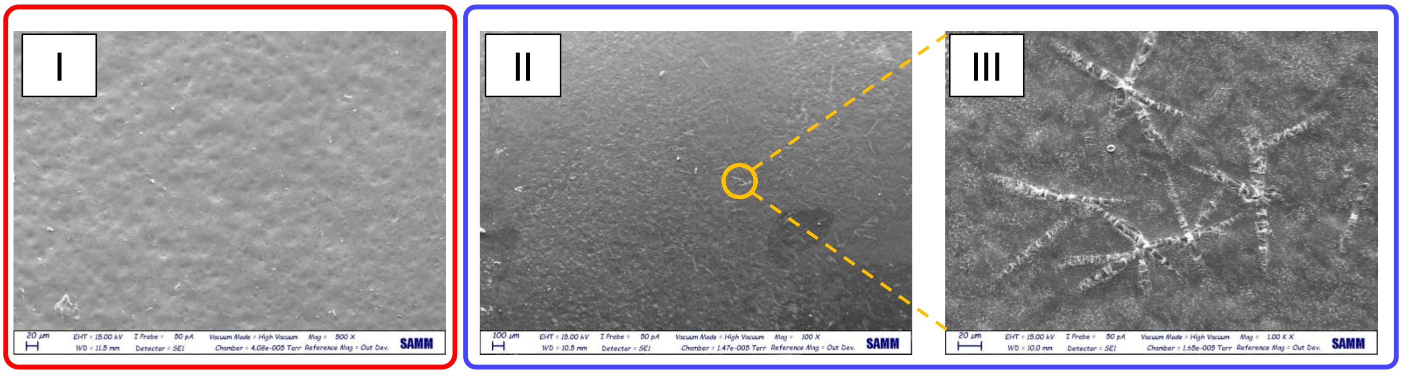

Figure S. 13 SEM micrographs of the top surfaces of blended films (BSF protein/PVOH/Glycerol, 1:1:1) obtained with (I, scale bar: 20µm) and without (II and III, scale bars: 100 and 20 µm, respectively) the fibrillization process.

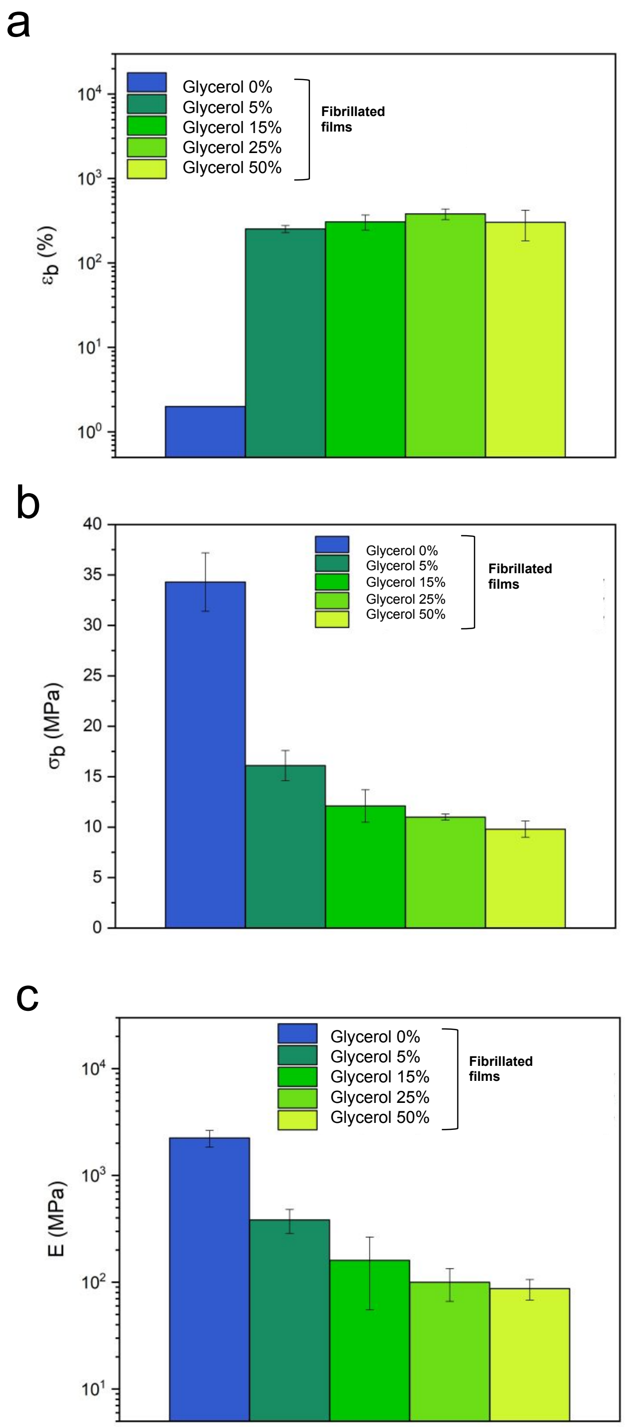

Figure S. 14 Tensile properties of fibrillated blended films at varying loadings of glycerol. (a) Elongation at break (ε_b) values; **(b)** Stress at break (σ_b) values; **(c)** Young's Modulus (E) values. Data are shown as mean ± SD (n=3).

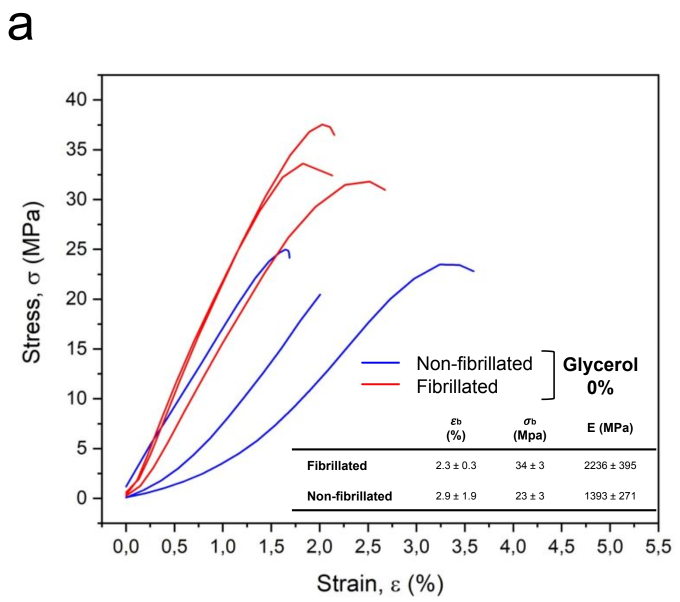
a
Stress, σ (MPa)
Strain, ε (%)
Non-fibrillated
Fibrillated
Glycerol
0%
εb (%)
σb (Mpa)
E (MPa)
Fibrillated 2.3 ± 0.3 34 ± 3 2236 ± 395
Non-fibrillated 2.9 ± 1.9 23 ± 3 1393 ± 271

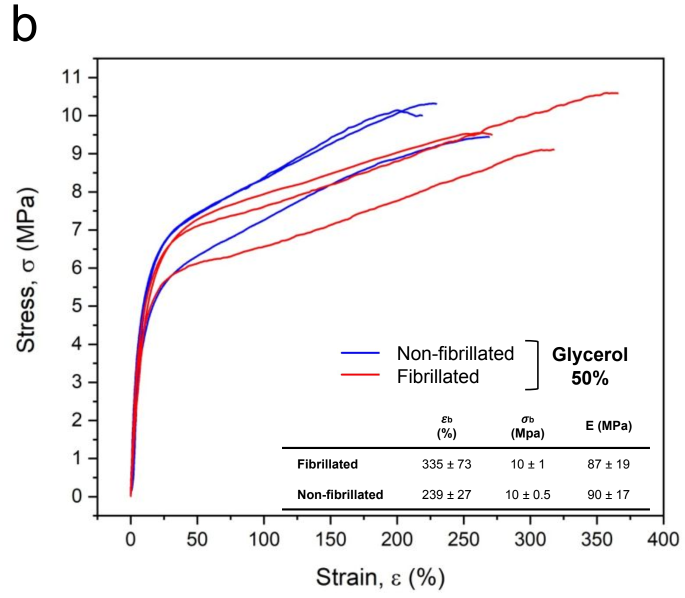
b
Stress, σ (MPa)
Strain, ε (%)
Non-fibrillated
Fibrillated
Glycerol
50%
εb (%)
σb (Mpa)
E (MPa)
Fibrillated 335 ± 73 10 ± 1 87 ± 19
Non-fibrillated 239 ± 27 10 ± 0.5 90 ± 17

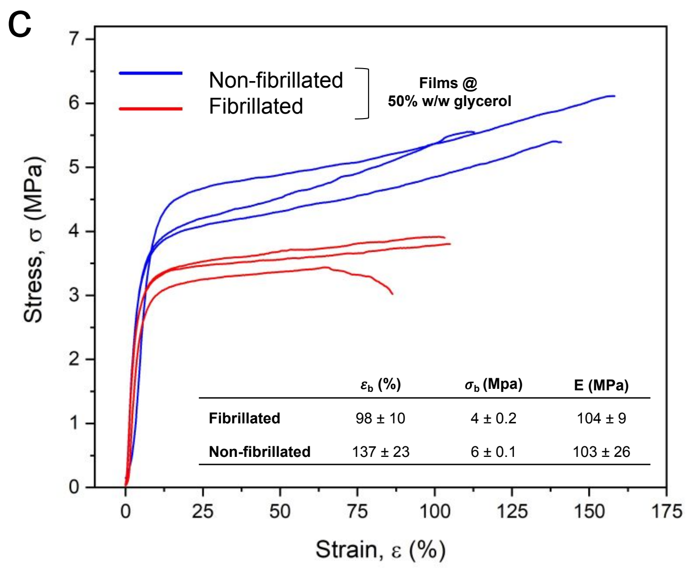

Figure S. 15 Stress-strain curves of fibrillated vs. non fibrillated films. **(a)** Blended films (BSF protein/PVOH/Glycerol, 1:1:0)**,** 0% w/w glycerol loading; **(b)** Blended films (BSF protein/PVOH/Glycerol, 1:1:1), 50% w/w glycerol loading; **(c)** Films without PVOH (BSF protein/Glycerol, 2:1)**,** 50% w/w glycerol loading. Tests were conducted in triplicate for each condition. The inset table reports the mechanical parameters associated with the graph. Data are reported as mean ± SD (n=3).

Table S. 4 Gas barrier properties of blended (BSF protein/PVOH) films. The table reports the O_2 (OTR) and CO_2 (CO_2TR) transmission rates and computed permeability coefficients (OP and CO_2P) for fibrillated and non-fibrillated blended films (50% w/w glycerol) determined under standard conditions (ASTM D3985: 23°C, RH 0%).

	**OTR**	**CO_2TR**	**OP**	**CO_2P**
	**(cm^3 m^{-2} d^{-1})**	**(cm^3 m^{-2} d^{-1})**	**(cm^3 µm m^{-2} d^{-1} Pa^{-1})**	**(cm^3 µm m^{-2} d^{-1} Pa^{-1})**
Non-fibrillated film	1.6	6.5	0.0012	0.0052
Fibrillated film	0.8	1.9	0.0008	0.0017

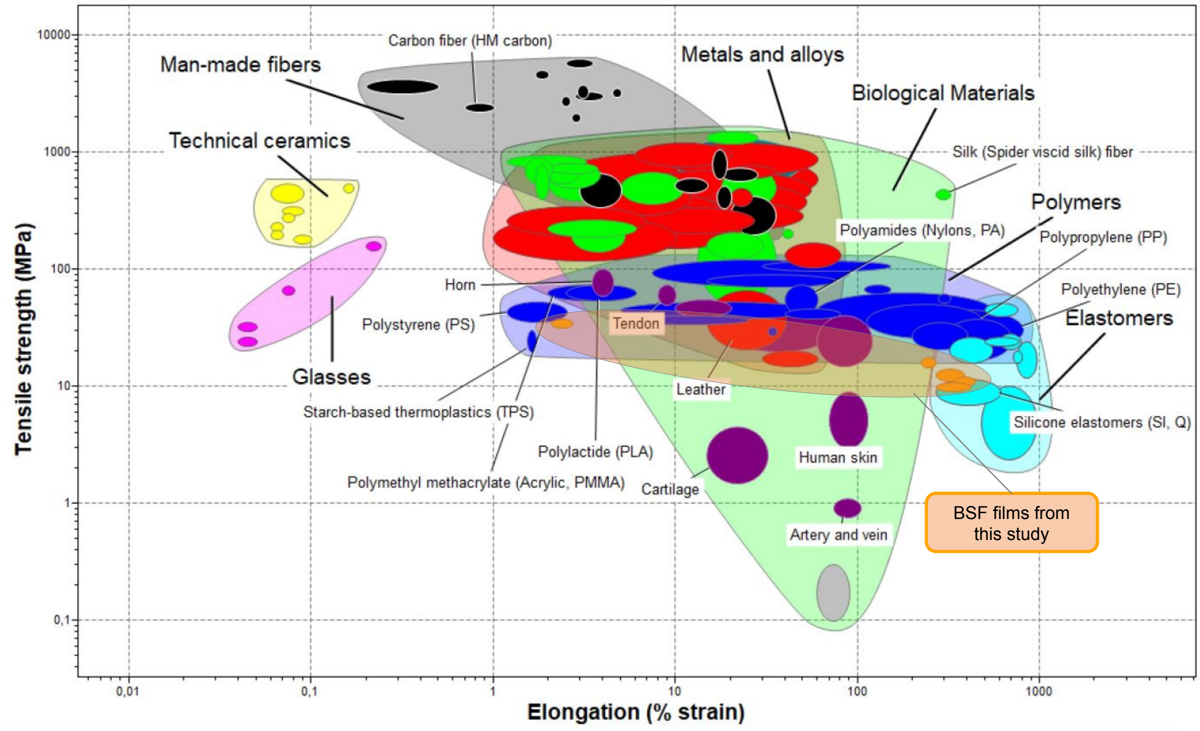

Figure S. 16. Tensile mechanical properties of blended films - Benchmark to other materials. Elongation (% strain) vs. Tensile strength (MPa). Data and graphs generated with the software Ansys Granta EduPack 2021.

Table S. 5 Oxygen permeability (OP) of main oil-based polymers and natural polymers.

Polymer Family	Polymer	OP* (mL µm m^{-2} d^{-1} Pa^{-1})	Ref.
Oil based polymers	Polyethylene terephthalate (PET)	0.015 – 0.05	
	Polypropilene (PP)	0.4 - 1	
	High-density polyethylene (HDPE)	0.6 – 0.7	
	Low-density polyethylene (LDPE)	1 – 4.4	
	Polystyrene (PS)	1 – 1.5	[55,57,58]
	Polyamide (PA)	0.001 – 0.014	
	Polyvinylalchol (PVOH)	0.0002 – 0.01	
	Polyvinylidene chloride (PVDC)	0.00012 - 0.003	
	Ethylene vinyl alcohol copolymer (EVOH)	0.00002 - 0.004	
Proteins	**BSF proteins/PVOH**	**0.0012**	**This study**
	Wheat gluten	0.002 - 2	[59,60]
	Gelatin	0.002 - 0.005	[61]
	Zein	0.04 - 0.25	[60,62]
	Whey Protein	0.05 - 0.1	[62,63]
	Silk Fibroin	0.16 - 6	[64]
Polysaccharides	Chitosan	0.02 - 1	[60,65]
	Hydroxypropyl Methyl Cellulose	0.3	[62]
	Methyl Cellulose	0.1 - 1.1	[60,62]
Lipids	Beeswax	0.05	[63]
	Candelilla Wax	0.08	[60]
	Shellac	0.8	[60]

* Measured at 23°C, RH 0-50%.
